# Impact of early life exposure to heat and cold on linguistic development in two-year-old children: findings from the ELFE cohort study

**DOI:** 10.1186/s12940-025-01173-8

**Published:** 2025-04-09

**Authors:** Guillaume Barbalat, Ariane Guilbert, Lucie Adelaïde, Marie-Aline Charles, Ian Hough, Ludivine Launay, Itai Kloog, Johanna Lepeule

**Affiliations:** 1 Team of Environmental Epidemiology Applied to Development and Respiratory Health, Institute for Advanced Biosciences (IAB), Université Grenoble Alpes, Inserm, CNRS, La Tronche, 38700 France; 2 Centre ressource de réhabilitation psychosociale et de remédiation cognitive, Pôle Centre rive gauche, Hôpital Le Vinatier, UMR 5229, CNRS & Université Claude Bernard Lyon 1, Lyon, France; 3 Santé publique France, 12 rue du Val d’Osne, Saint-Maurice Cedex, 94415 France; 4 Ined, Inserm, EFS, ELFE Joint Unit, Paris, France; 5 Université Grenoble Alpes, CNRS, INRAE, IRD, INP-G, IGE (UMR 5001), Grenoble, France; 6 U1086 Inserm Anticipe, Avenue Général Harris, Caen Cedex, 14076 France; 7 University Hospital of Caen, Caen Cedex, 14076 France; 8 Plateforme MapInMed, US PLATON, Avenue Général Harris, Caen Cedex, 14076 France; 9 The Department of Environmental, Geoinformatics and Urban Planning Sciences, Ben-Gurion University of the Negev, Beersheba, Israel; 10 Department of Environmental Medicine and Public Health, Icahn School of Medicine at Mount Sinai, New York, NY USA

**Keywords:** Temperature, Infants, Pregnancy, Language, Neurodevelopment

## Abstract

**Background:**

A number of negative developmental outcomes in response to extreme temperature have been documented. Yet, to our knowledge, environmental research has left the question of the effect of temperature on human neurodevelopment largely unexplored. Here, we aimed to investigate the effect of ambient temperature on linguistic development at the age of 2 years-old.

**Methods:**

We used data from the prospective national French birth cohort ELFE (*N* = 12,163) and highly-resolved exposure models with daily temporal resolution and 200 m to 1 km spatial resolution. We investigated the effect of weekly averages of overall, daytime and night-time temperature in the prenatal (first 30 weeks of gestation) and postnatal (91 weeks after birth) period on vocabulary production scores from the MacArthur-Bates Communicative Development Inventories (MB-CDI) at 2 years-old. Exposure-response and lag-response relationships were modeled with confounder-adjusted distributed lag non-linear models.

**Results:**

Scores at the MB-CDI decreased by 3.2% (relative risk (RR) 0.968, 95% confidence interval (CI): 0.939–0.998) following exposure to severe night-time heat of 15.6 °C (95th percentile) vs. 8.3 °C (median) throughout gestational weeks 14 to 19. In the postnatal period, scores at the MB-CDI decreased by 14.8% (RR 0.852; 95% CI: [0.756–0.96]) for severe overall heat of 21.9 °C (95th percentile) vs. 11.5 °C (median) throughout weeks 1 to 28. Consistent results were found for daytime and night-time heat. We observed positive effects of overall and night-time heat in the first few weeks of pregnancy. Night-time cold in the pre-natal period also resulted in improved scores at the MB-CDI. Adjusting our models for air pollutants (PM2.5, PM10 and NO2) tended to confirm these observations. Finally, there were no significant differences in temperature effects between boys and girls.

**Conclusion:**

In this large cohort study, we showed a negative impact of hot temperatures during pregnancy and after birth on language acquisition. Positive associations observed in the first few weeks of pregnancy are likely the results of methodological artifacts. Positive associations with night-time cold during the prenatal period are likely truly protective, as colder temperatures may encourage staying indoors at a comfortable temperature. Policymakers should consider neurodevelopment impairments as a deleterious effect of climate change.

**Supplementary information:**

The online version contains supplementary material available at 10.1186/s12940-025-01173-8.

## Background

The frequency and intensity of heatwaves and other extreme weather events is increasing rapidly and will continue to rise in the coming decades [1]. Extreme temperature is a major contributor to the global burden of disease, with high temperatures accounting for 156.81 Disability Adjusted Life Years (DALYs) per 100,000 population worldwide in 2019 [2]. Both pregnant women and infants are particularly susceptible to temperature stress, in part because thermoregulation processes are not as efficient during pregnancy [3] and infancy [4]. A number of negative developmental outcomes have been observed as a result of high temperatures, e.g. still-birth [5], prematurity [6], low birth weight [7, 8], altered lung function [9, 10], infections and allergies [11].

Above-average temperatures may also be associated with adverse neurodevelopmental outcomes. First, in animal experiments and in human studies, high temperatures have been shown to directly impact neurobiological mechanisms such as neuronal proliferation, differentiation and migration, as well as gliogenesis and myelination [12]. These in turn have been related to cognitive and behavioral impairments in the fish [13, 14]. Second, both heat [15, 16] and cold [17] have been associated with reduced weight and impaired functioning of the placenta, which may lead to further disruption of neurodevelopmental processes [18]. Third, heat may lead to a series of medical conditions, e.g. dehydration and decreased blood flow, low birth weight [7] or pre-eclampsia [19], which themselves have been linked to neurodevelopmental disorders [20, 21]. Fourth, exposure to heat during pregnancy has been associated with a higher rate of brain malformation and neural tube defects in human newborns, as well as psychiatric disorders diagnosed later in life [22], and reductions in academic achievement [23]. In utero exposure to heat has also been linked to a range of childhood and adult conditions, such as lack of cooperation [24], decreased well-being [25] and altered mental health [26].

Fifth, children [23, 27] and adult populations [28] have demonstrated cognitive dysfunctions, such as difficulties with attention-demanding tasks and decreased academic achievements, when acutely exposed to high temperatures. Acute exposures have also been linked to behavioral issues. Urban areas in the United States have seen a significant association between high minimum and maximum temperatures and crisis help-seeking behaviors in young adults and adolescents [29]. New York City data revealed that elevated temperature over the five preceeding days correlated with a higher risk of mental health-related emergency department and hospital encounters for 6- to 11-year-olds [30].

Sixth, longer-term temperature exposures have been associated with adverse neurodevelopmental outcomes. A Spanish study found that exposure to heat in the previous two months was associated with attention problems in adolescents [31]. A Californian study demonstrated a significant link between rising average temperatures over the preceding 1–3 years and increased aggressive behaviors in children aged 9–18 years [32]. To our knowledge however, no studies have investigated whether chronic temperature exposures in the post-partum period bear consequences for the neurodevelopment of the newborn.

Despite these notable advances, environmental research has left questions regarding the effect of temperature on certain aspects of human neurodevelopment largely unexplored, in particular aspects related to linguistic development. Language impairments are considered common reasons for referral in pediatrics [33] and have been linked with a range of negative outcomes. These include delayed academic achievement, increased risk of neurodevelopmental and psychiatric disorders, as well as behavioral and social issues [34, 35].

In the current study, we investigated the effect of ambient temperature on linguistic development at the age of two years old. First, we aimed to identify critical windows of exposure to heat and cold during prenatal and postnatal periods. Second, we assessed whether any effect of air temperature persisted independently of the influence of air pollutants. Third, we explored potential sex-specific associations. Sexual dimorphism in brain structure and function, as well as related neurodevelopmental issues, has been extensively documented [36, 37]. Previous research has demonstrated differential effects of temperature on health outcomes between boys and girls, including increased mortality risks during heatwaves [38], greater susceptibility to heat-related lung function declines [9], and heightened risks of preterm birth [7].

We used data from the large prospective French birth cohort ELFE [39] and highly-resolved temperature exposure models with one day temporal resolution and one km spatial resolution (200 m in large urban areas) [40].

## Methods

### The ELFE prospective birth cohort

We relied on the large French prospective birth cohort named Etude Longitudinale Française depuis l’Enfance (ELFE), which aims “to study determinants of the development health and socialization of children from birth to adulthood through a multidisciplinary approach” [39]. Briefly, the cohort was launched in 2011 in 344 maternity units in Metropolitan France drawn based on their size. Newborns were included over four waves across four periods 1–4 April, 27 June-4 July, 27 September-4 October, and 28 November-5 December. Exact home addresses (including changes of residence during pregnancy and childhood) were geocoded using a parcel-level database.

The cohort exclusion criteria were: multiple births of more than two children; underaged parents or parents who were not capable of giving informed consent; planning to leave metropolitan France within the next three years; inability to read/understand French, Arabic, Turkish or English; and children born before 32 weeks of amenorrhea. 51% of the mothers that met inclusion criteria agreed to participate and 18,329 children were enrolled. For the purpose of this study, we excluded mothers with multiple gestation (*N* = 576 children).

Demographic, socioeconomic and lifestyle information, as well as information on the child’s development were collected by questionnaire at each survey wave at the maternity unit (face to face interview with the mother), and during regular follow-ups of the children (telephone interview with both parents).

Informed consent was signed by the parents or the mother alone, with the father being informed of his right to deny the consent for his child’s participation. The ELFE study was approved by the Advisory Committee for Treatment of Health Research Information (Comite Consultatif sur le Traitement des Informations pour la Recherche en Sante), the National Data Protection Authority (CNIL) and the National Statistics Council.

### Assessment of linguistic development

Our outcome variable was the total score from the vocabulary production checklist of the MacArthur-Bates Communicative Development Inventories (MB-CDI). This was assessed by asking parents to state whether their two-year-old child is able to pronounce spontaneously each word out of a list of 100 French words. The MB-CDI has shown interesting psychometric properties such as internal and external consistency, test-retest reliability, convergent and concurrent validity as well as predictive validity [41].

Measures were taken between 23 and 28 months of age. Where values were missing at the mother’s interview, we used values reported by the father (if available). We then calculated the total score as the number of words correctly pronounced by the child. Higher scores indicate better linguistic development.

### Exposure to temperature

We estimated daily ambient temperature at mother’s and child’s home address using highly resolved spatiotemporal modeling [40]. The geospatial model used a multi-stage ensemble approach combining three basis learners (among which linear mixed models, random forests and gradient boosting) to calibrate temperature measured at monitoring stations with spatiotemporal predictors [40]. Predictions of ambient temperature were estimated from 2000 to 2018 at a 1 km spatial resolution across metropolitan France and at a 200 m spatial resolution over urban areas with > 50,000 inhabitants. Three indicators of temperature were calculated: minimum, maximum, and mean air temperature, markers of night-time, daytime and overall exposures, respectively. In terms of model performance, mean cross-validated R^2^ were higher than 0.9. For more in depth model descriptions please refer to [40].

We used both a prenatal and a postnatal exposure matrices to allow for different effect structures in the prenatal vs. the postnatal period. We ensured that the duration of exposure was consistent among all participants. We also decided to not exclude premature babies as this could introduce selection bias. Instead, we decided to include all live births from the ELFE cohort and restricted our prenatal period to 32 weeks of amenorrhea. With respect to the postnatal period, we reasoned that the last weeks of exposure would poorly contribute to processes involved in linguistic development. We therefore only considered the first 91 weeks of exposure (~ 21 months after birth).

### Covariates

Covariates were selected based on literature review and represented in a directed acyclic graph (DAG; Supplementary Fig. 1). A detailed description of our covariate selection process is provided in the Supplementary Methods. Briefly, the main covariates included variables related to the neighborhood’s socio-economic context and urbanization, vegetation, parental socio-economic status and demographic indicators, the number of languages spoken at home, pre-pregnancy medical history (such as parity, neurodevelopmental disorders, and maternal obesity), as well as the child’s age and sex. In our main analysis, we also incorporated various parental behaviors that may influence neurodevelopment, such as food intake, breastfeeding, alcohol consumption, and tobacco use during or after pregnancy (termed “Pre- and post-natal food/drug exposure” in our DAG; Supplementary Fig. 1A). These factors were included in our statistical model to enhance the precision of our parameter estimates. However, previous studies have suggested that climatic conditions may influence these behaviors (Supplementary Methods), which may then lie in the causal pathway between temperature and linguistic development (Supplementary Fig. 1B). To address this possibility, we conducted a sensitivity analysis where we did not adjust for such parental behavior covariates. This approach allowed us to assess the robustness of our results to the potential mediating role of these factors.

In our baseline analysis, we investigated the total effect of temperature on language development, not accounting for pollution. In a series of secondary analyses, we took into account atmospheric pollutants (PM2.5, PM10, NO2) in our statistical model to estimate the effect of temperature after ambient pollution has been explained away.

### Missing data

**Fig. 1:**
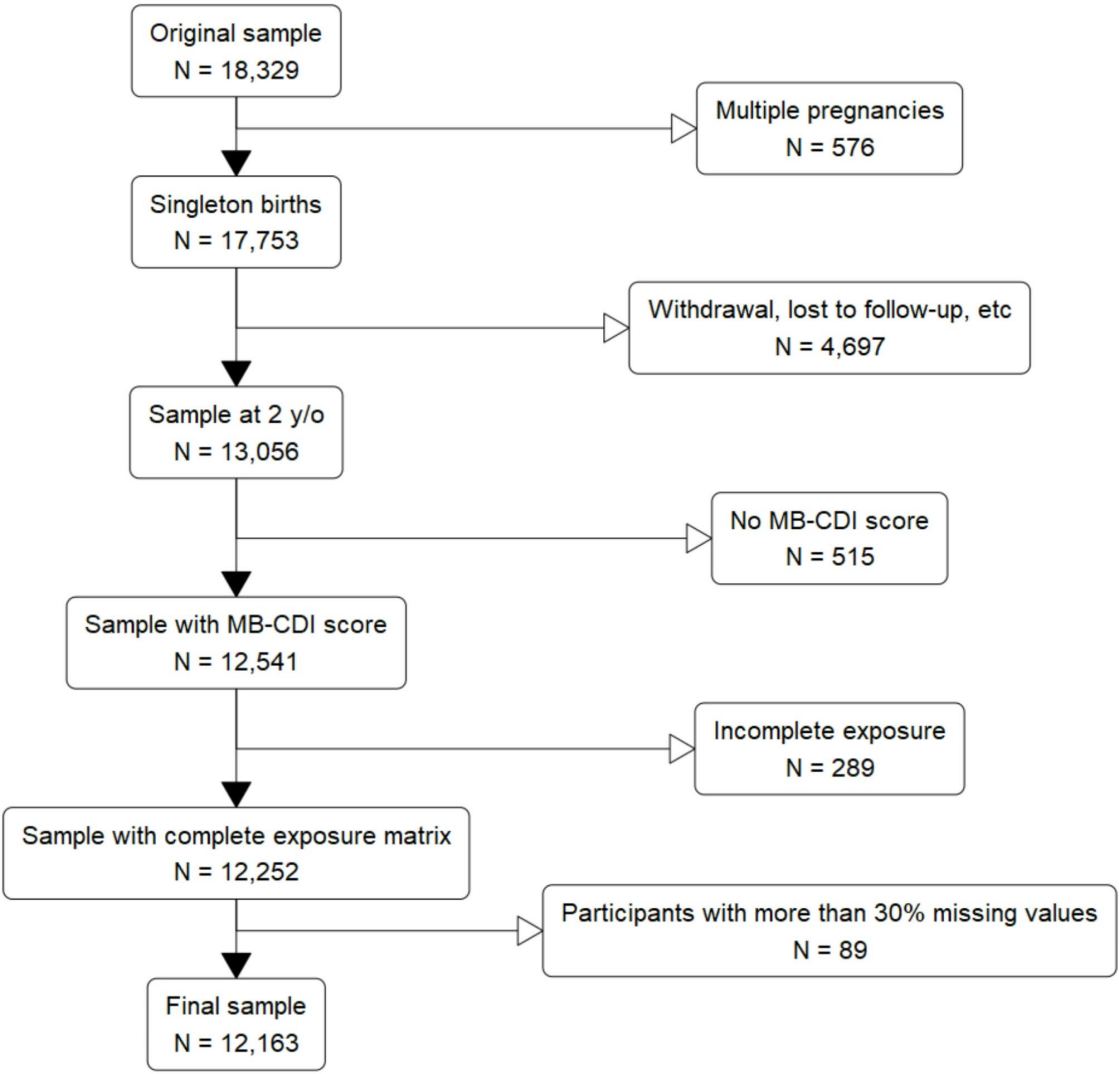
Flow chart of the study population Legend. MB-CDI, MacArthur-Bates Communicative Development Inventories; y/o, years old

From the original 17,753 single pregnancy observations, 4,697 were lost due to withdrawal or lost to follow-up. Among the remaining participants, we excluded 515 participants with missing values for the MB-CDI and 289 further participants with missing exposure data. There were 38 children whose parents were separated and had shared custody. For these children, we used the mother’s addresses to calculate exposures. After having removed participants with more than 30% missing values in the covariates, the final dataset comprised *N* = 12,163 participants (Fig. 1). Following this procedure, no covariate had more than 30% missing values.

To increase the statistical power of the analyses and avoid selection bias, missing data in the covariates were imputed with the *mice* R package (using single imputation and the predictive mean matching method). Imputation relied on prediction from mean exposures, covariates and outcome.

### Analysis

In order to identify sensitive windows of prenatal and postnatal exposure to temperature in relation to the vocabulary production score from the MB-CDI, we used the framework of distributed lag nonlinear models (DLNMs) and the *dlnm* R package [42]. With DLNMs, one can simultaneously represent non-linear exposure-response dependencies and delayed effects.

First, we modeled exposure to temperature with “cross-basis” functions, which describe simultaneously the exposure-response relationship and its distributed lag effects (effects across lags). As mentioned above, we used two exposure matrices: the 30 first weeks of pregnancy and the 91 weeks after birth. Both the exposure-response and the lag-response functions were assumed to be non-linear, with natural cubic splines. Optimal degrees of freedom were selected based on minimizing the Akaike Information Criterion (AIC) and on an a priori assumption of a relatively simple, yet flexible, association between temperature and linguistic development, as well as visual inspections of the lag-response relationships. Both prenatal and postnatal relationships were modeled using two degrees of freedom for exposure-response relationships and three degrees of freedom for lag-response relationships.

Second, we combined these cross-basis variables in generalized linear models of the quasi-Poisson family to evaluate exposure-lag-response associations between prenatal and postnatal exposure and scores on the MB-CDI.

In our main analysis, to estimate the total effect of temperature on the total score on the MB-CDI, we used the following equation:

1 $$\:\begin{array}{c}{Y}_{i}\text{~}Poisson\left({\lambda\:}_{i}\right)\\\:log\left({\lambda\:}_{i}\right)={\alpha\:}^{T}+{\beta\:}_{1}^{T}cb\left({Temp}_{{pre}_{i}}^{T}\right)\\+{\beta\:}_{2}^{T}cb\left({Temp}_{{post}_{i}}^{T}\right)+{\sum\:}_{k=1}^{n}{\gamma\:}_{k}^{T}{X}_{{k}_{i}}\end{array}$$

, where $\:{Y}_{i}$ is the score on the MB-CDI obtained by child $\:i$; $\:Poisson$ denotes the Poisson distribution; $\:{\lambda\:}_{i}$ is the expected MB-CDI score of child $\:i$, which is also equal to the variance. $\:{\lambda\:}_{i}$ is modeled using a log link function where $\:{\alpha\:}^{T}$ is the model’s intercept; $\:cb\left({Temp}_{{pre}_{i}}^{T}\right)$ and $\:cb\left({Temp}_{{post}_{i}}^{T}\right)$ are the cross-basis (pre- and postnatal) for temperature modality $\:T$ ($\:Tmin,Tmean,Tmax$) for child $\:i$ with corresponding set of coefficients $\:{\beta\:}_{1}^{T}$ and $\:{\beta\:}_{2}^{T}$; $\:{X}_{{k}_{i}}$ is the value of the covariate $\:k$ for child $\:i$ with corresponding coefficient $\:{\gamma\:}_{k}^{T}$.

Third, we used the fitted models to estimate the relative risk associated with exposure to cold and hot temperatures in a single week in both the prenatal and postnatal periods. Comparisons were made between moderate (10th, 90th percentiles), severe (5th, 95th percentiles), and extreme (1st, 99th percentiles) temperatures compared to the median temperatures. Of note, in a regression model of the quasi-Poisson family, the relative risk (RR) is interpreted as the multiplicative change in the rate of the outcome for a change in the predictor variable, holding other variables constant. In our case, a risk lower than one indicates that heat or cold, when compared to the median temperature, is associated with a percentage reduction in the MB-CDI score of $\:\left(1-RR\right)\ast\:100$. Conversely, a risk higher than one indicates that heat or cold, relative to the median temperature, is linked to a percentage increase in the MB-CDI score of $\:\left(RR-1\right)\ast\:100$.

We defined critical windows of exposure as consecutive periods where exposure risks were significant. The significant level was set to 0.05. Where critical windows were identified, we calculated the mean cumulative risk and 95% confidence interval associated with exposure throughout its entire duration.

Fourth, in a secondary analysis, we also modeled exposure to pollutants with pre- and postnatal cross-basis functions, where the exposure-response functions were assumed to be linear, and the lag-response relationships were assumed as varying smoothly across time (with natural cubic spline and three degrees of freedom).

To estimate the (direct) effect of temperature on the MB-CDI score over and above that of air pollution, we used the following equation:

2 $$\begin{array}{c}{Y}_{i}\text{~}Poisson\left({\lambda\:}_{i}\right)\\\:log\left({\lambda\:}_{i}\right)={\alpha\:}^{T,P}+{\beta\:}_{1}^{T,P}cb\left({Temp}_{{pre}_{i}}^{T}\right)+{\beta\:}_{2}^{T,P}cb\left({Temp}_{{post}_{i}}^{T}\right)\\+{\beta\:}_{3}^{T,P}cb\left({AP}_{{pre}_{i}}^{P}\right)+{\beta\:}_{4}^{T,P}cb\left({AP}_{{post}_{i}}^{P}\right)+{\sum\:}_{k=1}^{n}{\gamma\:}_{k}^{T,P}{X}_{{k}_{i}}\end{array}$$

, where $$\:cb\left({AP}_{{pre}_{i}}^{P}\right)$$ and $$\:cb\left({AP}_{{post}_{i}}^{P}\right)$$ are the cross-basis (pre- and postnatal) for air pollutant $$\:P$$$$\:\left(PM2.5,PM10,{NO}_{2}\right)$$, in models with temperature modality $$\:T$$$$\:Tmin,Tmean,Tmax$$$$\:i$$$$\:{\beta\:}_{3}^{T,P}$$ and $$\:{\beta\:}_{4}^{T,P}$$. Other terms are as in Eq. (1).

In another analysis, we wished to assess the robustness of our results by including more than one pollutant in the same statistical model. However, we deliberately chose not to run a model incorporating both PM2.5 and PM10 due to their high correlation (*r* > 0.9). This decision was made to avoid potential multicollinearity issues that could affect the reliability of our estimates.

Fifth, in each sex stratum, we identified critical windows and estimated cumulative risks with 95% confidence interval throughout their entire duration, similarly to our main analysis. Where critical windows were identified, statistical significance tests between boys and girls were conducted by calculating ratios of risk ratios $\:\frac{{RR}_{1}}{{RR}_{2}}$ as well as 95% confidence intervals using the following formula [43, 44]: $$\:{e}^{\begin{array}{c}log\left(\frac{{RR}_{1}}{{RR}_{2}}\right)\pm\:1.96\sqrt{{SE}_{log\left({RR}_{1}\right)}^{2}+{SE}_{log\left({RR}_{2}\right)}^{2}}\end{array}}$$, where $\:{RR}_{1}$ and $\:{RR}_{2}$ are the within-group estimates, and $\:{SE}_{log\left({RR}_{1}\right)}$ and $\:{SE}_{log\left({RR}_{2}\right)}$ are their respective standard errors.

Using the aforementioned formulae, we also estimated between-group differences at each lag and identified critical windows where consecutive lags showed significant differences between boys and girls. For each identified window, we then calculated cumulative risks with 95% confidence intervals for each sex stratum. Finally, we conducted statistical significance tests to assess between-group differences for each identified window.

## Results

### Population characteristics

About 20% of participants lived in rural areas or in urban areas with less than 50,000 inhabitants. Most individuals lived in urban areas with more than 500,000 inhabitants (45.6%). Most women were 25 to 35 years old (70%) and were multiparous (54%). Approximately one-third of women reported having had some exposure to smoking (active or passive) during pregnancy. Most of the time, at least one member of the couple had completed two years of post-secondary education (72%).

Overall, daytime and night-time mean temperatures experienced by participants were 12.7 °C (range: -11.6, 31.8), 18.1 °C (range: -8.5, 42.5), and 7.9 °C (range: -19.7, 26.5), respectively, in the prenatal period; and 12.0 °C (range: -20.1, 31.8), 17.0 °C (range: -15.7, 42.8), and 7.6 °C (range: -24.3, 29.1), respectively, in the postnatal period (Table 1; Supplementary Fig. 1). The median score on the MB-CDI was of 81 words (interquartile range: 59–93; Supplementary Fig. 2). Average exposure levels to PM2.5, PM10, and NO2 were of 15.3 µg/m³ (range: 0, 108), 22.5 µg/m³ (range: 0, 122), and 20.2 µg/m³ (range: 0, 238), respectively. Finally, comparing the imputed and non-imputed datasets indicated that the imputation process did not significantly change participants characteristics (Supplementary Table 1). Other characteristics of participants are reported in Table 1 and Supplementary Table 1.

**Table 1:** Socio-demographic characteristics of the study population

Variables	Total (*N* = 12163)
**MB-CDI Score**	
Median [Q1, Q3]	81 [59, 93]
**Daily overall temperature**^**a**^**(Tmean**,** °C)**	
Mean [min, max]	12.1 [-20.1, 31.8]
**Daily daytime temperature**^**a**^**(Tmax**,** °C)**	
Mean [min, max]	17.3 [-15.7, 42.8]
**Daily night-time temperature**^**a**^**(Tmin**,** °C)**	
Mean [min, max]	7.7 [-24.3, 29.1]
**Daily PM2.5 concentration** ^**a**^ **(µg/m³)**	
Mean [min, max]	15.3 [0, 108]
**Daily PM10 concentration** ^**a**^ **(µg/m³)**	
Mean [min, max]	22.5 [0, 122]
**Daily NO2 concentration** ^**a**^ **(µg/m³)**	
Mean [min, max]	20.2 [0, 238]
**European Defavor Index of living area**	
Low: (-9.18,-1.6]	4434 (36.5%)
Medium: (-1.6,2.06]	4194 (34.5%)
High: (2.06,31.9]	3519 (28.9%)
Missing	16 (0.1%)
**NDVI** ^**b**^	
Low: (0.0367,0.393]	3732 (30.7%)
Medium: (0.393,0.51]	4066 (33.4%)
High: (0.51,0.89]	4362 (35.9%)
Missing	3 (0.0%)
**Size of living area**	
Rural area or less than 50,000 inhabitants	2447 (20.1%)
50,000 to 500,000 inhabitants	3778 (31.1%)
More than 500,000 inhabitants	5543 (45.6%)
Missing	395 (3.2%)
**Mother’s birth place**	
France	10,903 (89.6%)
Overseas	1185 (9.7%)
Missing	75 (0.6%)
**Mother’s history of learning difficulties**	
No	6715 (55.2%)
Yes	4978 (40.9%)
Missing	470 (3.9%)
**Father’s history of learning difficulties**	
No	4964 (40.8%)
Yes	5017 (41.2%)
Missing	2182 (17.9%)
**Level of education (Highest between mother and father)**	
Primary or secondary school	264 (2.2%)
Highschool	2931 (24.1%)
Undergraduate	2675 (22.0%)
Bachelor	2191 (18.0%)
Postgraduate	3914 (32.2%)
Missing	188 (1.5%)
**Mother’s socio-professional category**	
Craftsmen & merchants	1010 (8.3%)
Executives and higher intellectual professions	3011 (24.8%)
Intermediate professions	1739 (14.3%)
Employees (skilled and unskilled)	4234 (34.8%)
Workers (skilled and unskilled)	1377 (11.3%)
Others	532 (4.4%)
Missing	260 (2.1%)
**Parity**	
Primiparous	5531 (45.5%)
Multiparous	6586 (54.1%)
Missing	46 (0.4%)
**Pre-pregnancy Body Mass Index**	
< 18.5	868 (7.1%)
18.5–25	8053 (66.2%)
25–30	2010 (16.5%)
> 30	1120 (9.2%)
Missing	112 (0.9%)
**Mother’s age at conception**	
25 or below	1139 (9.4%)
26–30	3988 (32.8%)
31–35	4521 (37.2%)
36–40	2046 (16.8%)
41 or above	453 (3.7%)
Missing	16 (0.1%)
**Father’s age at conception**	
25 or below	554 (4.6%)
26–30	2730 (22.4%)
31–35	4332 (35.6%)
36–40	2693 (22.1%)
41–45	1048 (8.6%)
46 or above	463 (3.8%)
Missing	343 (2.8%)
**Alcohol consumption during pregnancy**	
At least once a month	3128 (25.7%)
Never or less than once a month	8955 (73.6%)
Missing	80 (0.7%)
**Consumption of tobacco during pregnancy**	
At least some exposure (passive or active)	3844 (31.6%)
No exposure	7968 (65.5%)
Missing	351 (2.9%)
**Coffee consumption during pregnancy**	
Less than once a day	9966 (81.9%)
Once a day or more	1030 (8.5%)
Missing	1167 (9.6%)
**Fish consumption during pregnancy**	
Never	613 (5.0%)
Less than once a month	1313 (10.8%)
One to three times a month	3294 (27.1%)
Once a week	3714 (30.5%)
Twice a week or more	2160 (17.8%)
Missing	1069 (8.8%)
**Vitamin B9 consumption during pregnancy**	
No	5250 (43.2%)
Yes	6634 (54.5%)
Missing	279 (2.3%)
**Omega 3 consumption during pregnancy**	
Less than once a week	2567 (21.1%)
More than once a week	1796 (14.8%)
Never	5792 (47.6%)
Missing	2008 (16.5%)
**Sex of the child**	
Female	5988 (49.2%)
Male	6175 (50.8%)
**Feeding method at 2 months**	
Breastfeeing only	3963 (32.6%)
Breastfeeing & Bottle feeding	1927 (15.8%)
Bottle feeding only	5987 (49.2%)
Missing	286 (2.4%)
**Parental relationship**	
Separated	690 (5.7%)
Together	11,372 (93.5%)
Missing	101 (0.8%)
**Number of languages spoken at home**	
One	8566 (70.4%)
Two	2525 (20.8%)
Three or more	834 (6.9%)
Missing	238 (2.0%)
**Household income**	
1st quintile	2168 (17.8%)
2nd quintile	2349 (19.3%)
3rd quintile	2286 (18.8%)
4th quintile	2353 (19.3%)
5th quintile	2365 (19.4%)
Missing	642 (5.3%)
**Age at MB-CDI test (month)**	
Mean [min, max]	25.33 [23.00, 28.00]
Missing	101 (0.8%)

^a^ from conception to MB-CDI test

Legend. NDVI, Normalized difference vegetation index; MB-CDI, MacArthur-Bates Communicative Development Inventories; Q1, 1st quartile; Q3, 3rd quartile

### Ambient temperature and linguistic development at two years old (Non stratified analysis)

#### Heat

**Fig. 2:**
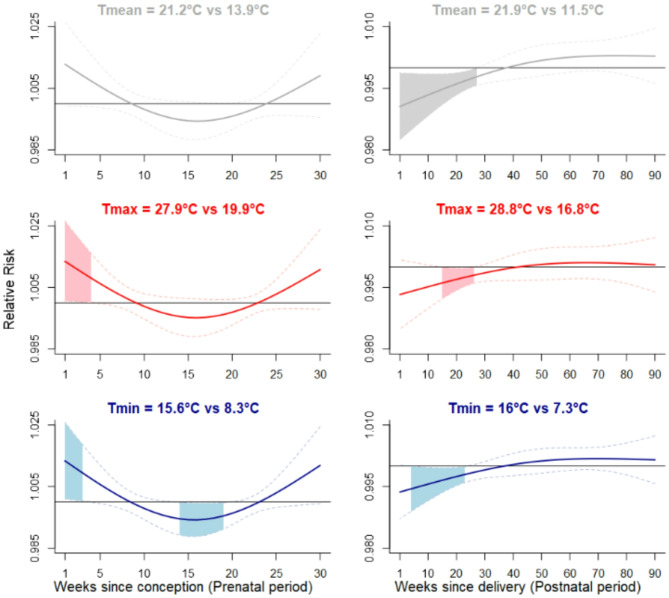
Lag-specific effects of Heat on the MB-CDI score (from models unadjusted for pollution) Adjusted relative risk (solid line) and 95% confidence interval (dashed lines) for the MB-CDI score associated with severe heat (95th percentile vs. 50th percentile) during the 30 weeks following conception (left) and the 91 first weeks of life (right). Risks lower than one indicate that higher temperatures, compared to the median temperature, are associated with a reduction in the MB-CDI score. Conversely, risks higher than one indicate that higher temperatures, compared to the median, are associated with an increase in the MB-CDI score Shaded areas indicate 95% confidence intervals that exclude one Upper panel: Overall temperature (Tmean); Middle panel: Daytime temperature (Tmax); Lower panel: Night-time temperature (Tmin) Legend. MB-CDI, MacArthur-Bates Communicative Development Inventories

In the prenatal period, while overall and daytime heat were not significantly associated with a decreased score at the MB-CDI, night-time heat was (Fig. 2). Specifically, throughout gestational weeks 14 to 19, scores at the MB-CDI decreased by 3.2% (Relative Risk (RR): 0.968; 95% Confidence Interval (CI): 0.939–0.998) following exposure to severe night-time heat (Tmin = 15.6 °C vs. Tmin = 8.3 °C), given the other variables in the model are held constant (Table 2). Consistent negative associations were observed for moderate (Tmin = 14.3°C) and extreme night-time heat (Tmin = 18.3°C; Table 2). Improved scores at the MB-CDI were found for daytime and night-time heat in the first three or four few weeks of pregnancy (Fig. 2; Table 2).

**Table 2:** Association of prenatal and postnatal cumulative exposure to heat and cold with the MB-CDI score (from models unadjusted for pollution)

	Prenatal period		Postnatal period	
Exposure	Temperature	Cumulative effect	Temperature	Cumulative effect
***Overall Heat***	*Ref (50th pct): Tmean = 13.9 °C*		*Ref (50th pct): Tmean = 11.5 °C*	
Moderate (90th pct)	Tmean = 19.8 °C	No critical window	Tmean = 20.0 °C	Wk 1–28: 0.883 (0.805–0.968)
Severe (95th pct)	Tmean = 21.2 °C	No critical window	Tmean = 21.9 °C	Wk 1–28: 0.852 (0.756–0.960)
Extreme (99th pct)	Tmean = 23.8 °C	No critical window	Tmean = 24.8 °C	Wk 1–28: 0.801 (0.678–0.947)
***Daytime Heat***	*Ref (50th pct): Tmax = 19.9 °C*		*Ref (50th pct): Tmax = 16.8 °C*	
Moderate (90th pct)	Tmax = 26.2 °C	Wk 1–3: 1.026 (1.001–1.052)	Tmax = 25.6 °C	Wk 16–27: 0.973 (0.951–0.996)
Severe (95th pct)	Tmax = 27.9 °C	Wk 1–4: 1.043 (1.001–1.087)	Tmax = 28.8 °C	Wk 16–27: 0.965 (0.935–0.996)
Extreme (99th pct)	Tmax = 30.7 °C	Wk 1–4: 1.065 (1.003–1.13)	Tmax = 32.1 °C	Wk 17–27: 0.957 (0.920–0.996)
***Night-time Heat***	*Ref (50th pct): Tmin = 8.3 °C*		*Ref (50th pct): Tmin = 7.3 °C*	
Moderate (90th pct)	Tmin = 14.3 °C	Wk 1–3: 1.027 (1.001–1.053) & Wk 13–20: 0.967 (0.939–0.997)	Tmin = 14.5 °C	Wk 1–24: 0.922 (0.859–0.989)
Severe (95th pct)	Tmin = 15.6 °C	Wk 1–3: 1.034 (1.002–1.068) & Wk 14–19: 0.968 (0.939–0.998)	Tmin = 16.0 °C	Wk 5–24: 0.927 (0.867–0.992)
Extreme (99th pct)	Tmin = 18.3 °C	Wk 1–4: 1.062 (1.004–1.124) & Wk 15–18: 0.969 (0.940–0.999)	Tmin = 18.9 °C	Wk 12–24: 0.946 (0.898–0.996)
***Night-time Cold***	*Ref (50th pct): Tmin = 8.3 °C*		*Ref (50th pct): Tmin = 7.3 °C*	
Moderate (10th pct)	Tmin = 0.1 °C	Wk 16–22: 1.033 (1.007–1.059)	Tmin = 0.2 °C	No critical window
Severe (5th pct)	Tmin=-1.5 °C	Wk 17–22: 1.033 (1.006–1.061)	Tmin=-1.8 °C	No critical window
Extreme (1st pct)	Tmin=-4.0 °C	Wk 18–22: 1.035 (1.004–1.066)	Tmin=-7.3 °C	No critical window

Cumulative adjusted risk ratio associated with ambient temperature exposure throughout an entire critical window for moderate, severe and extreme exposures. 95% confidence interval are also reported. Risks lower than one indicate that heat or cold, when compared to the median temperature, are associated with a reduction in the MB-CDI score. Conversely, risks higher than one indicate that heat or cold, relative to the median temperature, are linked to an increase in the MB-CDI score

Legend. MB-CDI, MacArthur-Bates Communicative Development Inventories; pct, percentile; Wk, Week

In the postnatal period, overall, daytime, and night-time heat were associated with lower scores at the MB-CDI (Fig. 2). Specifically, scores at the MB-CDI decreased by 14.8% (RR: 0.852; 95% CI: 0.756–0.960) following exposure to severe overall heat (Tmean = 21.9 °C vs. 11.5 °C) throughout weeks 1 to 28; by 3.5% (RR: 0.965; 95% CI: 0.935–0.996) following exposure to severe daytime heat (Tmax = 28.8 °C vs. 16.8 °C) throughout weeks 16 to 27; and by 7.3% (RR: 0.927; 95% CI: 0.867–0.992) following exposure to severe night-time heat (Tmin = 16 °C vs. 7.3 °C) throughout weeks 5 to 24 (Table 2). Consistent negative associations were observed for moderate and extreme heat (Table 2).

#### Cold

**Fig. 3:**
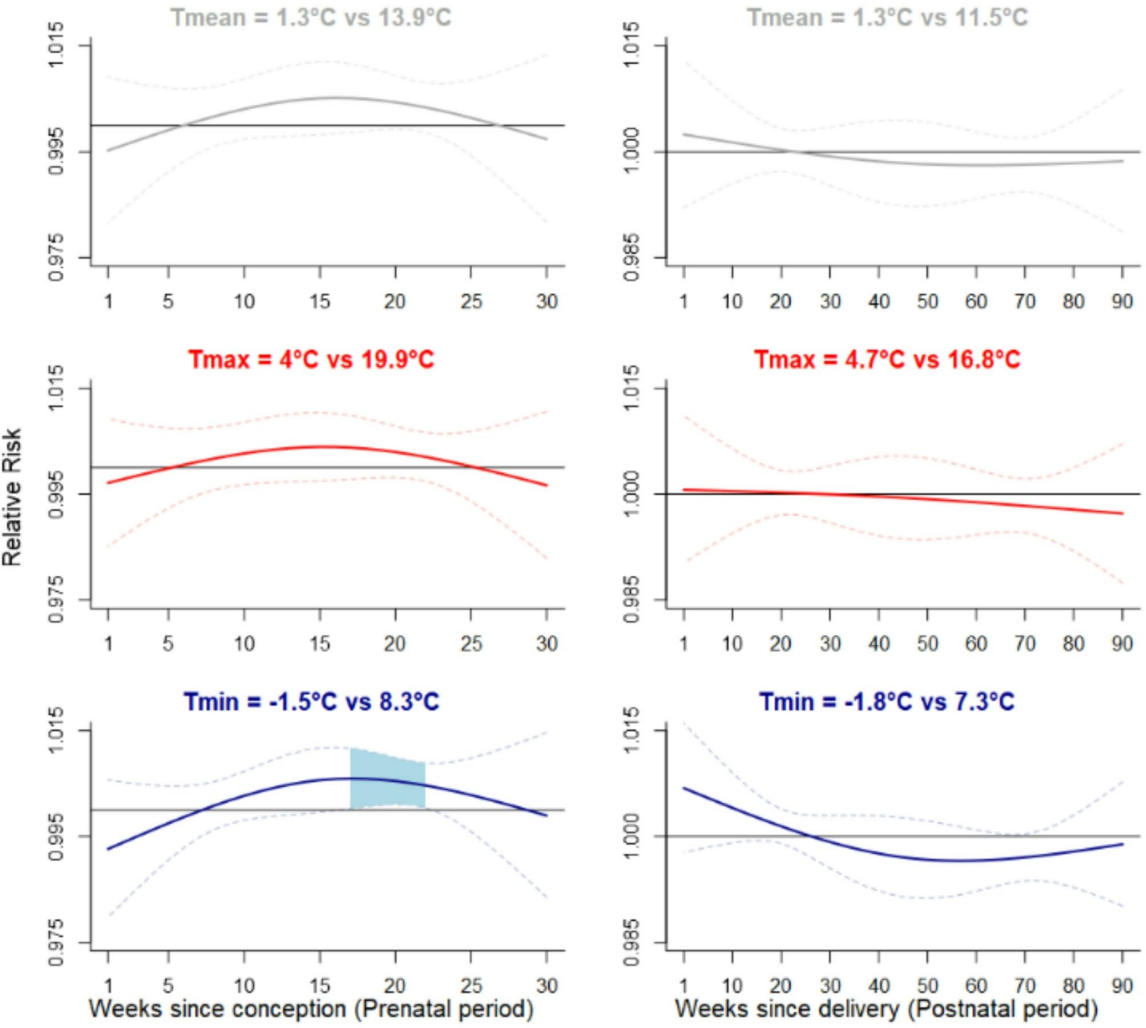
Lag-specific effects of Cold on the MB-CDI score (from models unadjusted for pollution) Adjusted relative risk (solid line) and 95% confidence interval (dashed lines) for the MB-CDI score associated with severe cold (5th percentile vs. 50th percentile) during the 30 weeks following conception (left) and the 91 first weeks of life (right). Risks lower than one indicate that colder temperatures, compared to the median temperature, are associated with a reduction in the MB-CDI score. Conversely, risks higher than one indicate that colder temperatures, compared to the median, are associated with an increase in the MB-CDI score Shaded areas indicate 95% confidence intervals that exclude one Upper panel: Overall temperature (Tmean); Middle panel: Daytime temperature (Tmax); Lower panel: Night-time temperature (Tmin) Legend. MB-CDI, MacArthur-Bates Communicative Development Inventories

In the prenatal period, overall, daytime, and night-time cold were not associated with lower scores at the MB-CDI (Fig. 3). However, improved scores at the MB-CDI were found for night-time cold towards the end of the second trimester (Fig. 3). Throughout gestational weeks 17–22, severe night-time cold (Tmin=-1.5 °C) resulted in an improvement in the MB-CDI score of 3.3% (RR: 1.033; 95% CI: 1.006–1.061). Consistent results were found for moderate and extreme night-time cold (Table 2).

In the postnatal period, overall, daytime, and night-time cold were not associated with any deteriorated or improved scores at the MB-CDI (Fig. 3).

Finally, our findings remained largely consistent when excluding parental behaviors that could potentially mediate the relationship between air temperature and neurodevelopment (Supplementary Table 2).

#### Adjusting on pollution

In general, the shapes of the lag-response curves obtained in our secondary analyses adjusting for PM2.5, PM10 and NO2 were similar to those from our main analyses (Supplementary Figs. 4 to 9).

Critical windows were also similar (Supplementary Table 3). Specifically, significant negative effects of severe night-time heat in the prenatal period were replicated when adjusting for PM10 and NO2. Significant negative effects of severe overall, daytime and night-time heat in the postnatal period were replicated when adjusting for NO2. Models adjusting for PM2.5 and PM10 showed significant negative effects of severe overall heat in the postnatal period. Significant positive effects of severe night-time cold in the prenatal period were observed when adjusting for PM2.5, PM10, and NO2. These results were confirmed when including more than one pollutant in the same model (Supplementary Table 3).

### Sex-stratified analysis

In each group, critical windows were identified and are reported in Supplementary Table 4. However, none of the identified windows showed significant differences between boys and girls (Supplementary Table 4). Our between-group analysis at each lag did not reveal any significant differences (Supplementary Figs. 10 & 11).

## Discussion

In the current study, night-time heat in the second trimester of pregnancy and overall, daytime and night-time heat in the first seven months after birth were associated with decreased scores in vocabulary production from the MB-CDI at the age of two. Unexpected positive effects of heat and cold on linguistic development were also reported. Most effects persisted after adjusting on PM2.5, PM10 and NO2. Finally, in our sex-stratified analyses, within-group differences were observed, but we did not identify statistically significant between-group differences.

Exposure to severe overall heat during the first seven months of postnatal life was associated with a 15% decrease in the MB-CDI score, which is a rather sizable effect. Apart from this, most cumulative percentage changes throughout the entire duration of a critical window of exposure were 8% or less. These findings may have significant public health implications, as climate change is expected to increase exposure to high temperatures, suggesting that targeted heat protection strategies during critical developmental periods– particularly the second trimester of pregnancy and early postnatal months– could potentially mitigate adverse impacts on neurodevelopment.

Several mechanisms may explain how temperature affects linguistic development. Neurobiological processes, such as impaired neuronal migration, neurite extension, or placental dysfunctions, may be at stake [12, 17]. Alternatively, the effect of temperature may be mediated by medical conditions such as pre-eclampsia, low birth weight, vaginal infections due to changes in the microbiome [8, 22] or even maternal psychological stress [22]. Other factors, such as availability of food and of basic nutrient intake, may play a role [8].

The fact that some of the effects found in our research persisted after adjusting our models on three different pollutants– PM2.5, PM10 and NO2– thought to be implied in cognitive development [45] may also have important public health implications. Because concentrations of ambient pollutants are thought to be influenced by air temperature, our results suggest that temperature has a direct effect on linguistic development, above and beyond that of air pollution. Therefore, to prevent neurodevelopmental impairment, environmental policymakers should focus on developing solutions that address both heat exposure and air pollution, rather than air pollution alone. Another important implication of this finding is that temperature should be considered as a confounder in studies investigating the impact of pollution on neurodevelopment, which to our knowledge has not been done consistently.

Unexpected, positive effects of heat were retrieved in the first trimester of pregnancy. One possibility is that heat is protective of neurodevelopment at the beginning of pregnancy, a rather counter-intuitive hypothesis. Another, perhaps more plausible hypothesis, is that such an effect occurred by chance, as it was observed at the very beginning of the lag-response curve, where confidence intervals are the greatest. As a third possibility, this effect may be attributed to a so-called live-birth bias [46]. In our study, an unmeasured factor may have impacted both fetal survival and neurodevelopmental outcomes. Because temperature is also responsible for fetal survival [8], selecting to live-birth observations would have artificially linked temperature to neurodevelopmental outcomes. Fourth, a study using data from sub-Saharan Africa reported that increased ambient temperatures during the first trimester were associated with higher educational attainment and literacy rates [47]. The authors proposed two potential mechanisms for this effect: (1) heat-induced selective pressure on fetal development, where elevated temperatures may disproportionately affect more vulnerable fetuses; and (2) heat-induced changes in conception patterns, particularly among economically disadvantaged women, potentially resulting in births occurring in more favorable socioeconomic conditions.

Though occurring later in pregnancy, the positive effect of night-time cold around weeks 15–23 may also reflect a live-birth bias. It is possible however that night-time cold is truly protective towards neurodevelopment, and that for at least four reasons. First, this effect incurred a relatively small confidence interval, hence is less likely to be due to chance alone. Second, we observed negative effects of night-time heat over a relatively similar period. Third, other studies have reported a protective effect of cold during pregnancy, e.g. on pre-term birth [48, 49]. Fourth, night-time cold outdoor temperatures are likely associated with staying indoors at an optimal temperature, particularly in affluent countries like France.

Comparison with the literature is difficult, first because not much data are available, second because populations, outcomes and methods of analysis are drastically different. We found a meta-analysis demonstrating that hot temperatures during pregnancy may be responsible for neural tube defects or psychiatric disorders presenting in later life, such as schizophrenia and restrictive anorexia [22]. A recent study also showed that exposure to both heat and cold during various periods of pregnancy and childhood was associated with reduced myelination and maturation of white matter microstructure [50]. Overall, we hope that further analysis testing the impact of temperature on neurodevelopment will emerge from our research.

In the current study, the critical period for the effect of heat in the prenatal period was found throughout the first few weeks of the second trimester. It is noteworthy that a recent review associating high ambient heat with “mental deficiency” and schizophrenia retrieved a rather similar critical window (end of the first trimester) [22]. This period is thought to be crucial for glial cell proliferation [12] and white matter development [51, 52], two processes that may explain impairments in language production in our study. Prenatal associations were stronger for night-time heat but the shape of the lag-response curve was similar for overall and daytime heat. As suggested by others, effects of heat may be more prominent at night as heat at night prevents the human body from recovering from daytime heat exposure [53, 54].

In our study, critical periods for linguistic development were not limited to the prenatal period. Heat effects were also retrieved in the first seven months after birth. Synaptogenesis, gliogenesis and apoptosis may be at stake as they carry on after birth, leading to structural and functional brain changes that are important for language acquisition [55]. A recent study in the rat also demonstrated that postnatal processes such as delayed myelinogenesis, neuroinflammation, oxydative stress, and disruption in gut microbiome, may impact neurodevelopment more prominently than in the prenatal period [56]. Larger effects in the postnatal vs. prenatal period may be due to the smaller thermoregulation abilities in infants compared to pregnant women [3].

Previous analyses have suggested that negative associations between ambient temperature in the prenatal period and neurodevelopmental disorders [22], or other health outcomes (e.g. birth weight [7]) may be sex-specific. Our results were not consistent with these findings. Various critical windows showing negative effects of temperature were identified during the postnatal period in each sex stratum but between-group differences did not pass statistical significance. Our sub-group analyses may have lacked power to identify subtle differences. Alternatively, different mechanisms may compensate one another, for instance increased efficiency of male fetus’ placentas may compensate for their increased susceptibility to various stressors [57]. Likewise, male fetuses have been reported to be at elevated risk of still-birth [58]. This may sign increased vulnerability but on the other hand, surviving male fetuses may be those who are more resilient to temperature stress.

### Strengths and limitations

First, one major strength of this study is the good performance of the fine spatial and temporal resolution of the exposure model, which likely decreased measurement error. Second, the use of weekly exposure lags may have assessed health effects of temperature with more precision than approaches where exposures are averaged over trimesters or months. Such rather long exposures risk diluting extreme temperature events and may bias health effects towards the null. Third, our modeling strategy, employing distributed lag models, enabled the identification of critical temperature exposure windows during both prenatal and postnatal development, revealing key periods of neurodevelopmental sensitivity. Fourth, our national study population benefited from a large number of observations and from being representative of the diverse climatic conditions across France.

Our study also has a number of limitations. First, we assessed outdoor temperature which may strongly differ from temperatures really experienced by participants. While we accounted for changes in residence, exposure misclassification may have occurred because women and children spent time indoors or out of town, or used heating or air conditioning devices– although the percentage of homes equipped with air conditioning in France is relatively low [59]. In general, there is a tradeoff between using large cohorts with the potential for exposure misclassification vs. small scale experiments that can measure exposures more precisely but have a limited number of participants, with the risk of low statistical power and poor generalizability.

Second, selection bias is a possibility, (1) because we excluded observations with missing exposure and outcome values; (2) because we restricted our analysis to live-birth observations.

Third, in this prospective longitudinal cohort study, residual confounding is a possibility.

Fourth, rather than comparing extreme temperatures with median temperatures, we could have defined our exposure differently, e.g. as 1 °C increment or even as heatwaves. We reasoned that neurodevelopment would be less sensitive to 1 °C increments than temperature threshold cutoff points. In addition, we can only infer that temperatures higher than the ones measured in the current study would have at least similar, and more probably worse impacts on neurodevelopment. This is particularly important in our case because, since 2003, years 2010 and 2011 were not particularly warm [40].

Fifth, it is important to acknowledge that some of our findings may in fact be falsely positive as some of the confidence intervals were relatively close to including one.

Sixth, the current results are probably not generalizable to non-Western countries located in warmer or colder regions, where acclimatization processes may be very different and result in different vulnerabilities to heat and cold.

## Conclusion

With global climate change, the impact of climate extremes on children’s health is only expected to increase. Our study revealed for the first time that severe heat (95th percentile) during both the prenatal and the postnatal periods might affect language production at the age of two. Sex differences however, were not identified. Should these findings be replicated, climate mitigation strategies would be beneficial to prevent issues in linguistic development.

## Electronic supplementary material

Below is the link to the electronic supplementary material.

Supplementary Material 1

## Data Availability

The data that support the findings of this study are not publicly available due to containing information that could compromise research participant privacy/consent. Further inquiries about the data used in this study can be directed to the corresponding author. Code used to analyse the data is available upon request due to the corresponding author.

## References

[1] IPCC. AR6 Synthesis Report. Climate Change [cited 2024 May 21]. 2023. Available from: https://www.ipcc.ch/report/sixth-assessment-report-cycle/

[2] Song J, Pan R, Yi W, Wei Q, Qin W, Song S et al. Ambient high temperature exposure and global disease burden during 1990–2019: An analysis of the Global Burden of Disease Study 2019. Science of The Total Environment. [cited 2024 Feb 8]. 2021;787:147540. Available from: https://www.sciencedirect.com/science/article/pii/S0048969721026115 10.1016/j.scitotenv.2021.147540 33992940

[3] Smith CJ. Pediatric Thermoregulation: Considerations in the Face of Global Climate Change. Nutrients. [cited 2024 Apr 23]. 2019;11:2010. Available from: https://www.ncbi.nlm.nih.gov/pmc/articles/PMC6770410/10.3390/nu11092010 PMC6770410 31454933

[4] Tsuzuki-Hayakawa K, Tochihara Y, Ohnaka T. Thermoregulation during heat exposure of young children compared to their mothers. Eur J Appl Physiol. 1995 [cited 2024 Apr 29];72:12–7. Available from: 10.1007/BF00964108 10.1007/BF00964108 8789564

[5] Bruckner TA, Modin B, Vågerö D. Cold ambient temperature in utero and birth outcomes in Uppsala, Sweden, 1915–1929. Annals of Epidemiology. 2014 [cited 2024 Jan 24];24:116–21. Available from: https://www.sciencedirect.com/science/article/pii/S1047279713004213 10.1016/j.annepidem.2013.11.005 24332864

[6] Hough I, Rolland M, Guilbert A, Seyve E, Heude B, Slama R, et al. Early delivery following chronic and acute ambient temperature exposure: a comprehensive survival approach. Int J Epidemiol. 2023;52:761–73. 36274245 10.1093/ije/dyac190

[7] Jakpor O, Chevrier C, Kloog I, Benmerad M, Giorgis-Allemand L, Cordier S, et al. Term birthweight and critical windows of prenatal exposure to average meteorological conditions and meteorological variability. Environ Int. 2020;142:105847. 32559561 10.1016/j.envint.2020.105847

[8] Chersich MF, Pham MD, Areal A, Haghighi MM, Manyuchi A, Swift CP et al. Associations between high temperatures in pregnancy and risk of preterm birth, low birth weight, and stillbirths: systematic review and meta-analysis. BMJ. 2020 [cited 2024 Apr 28];371:m3811. Available from: https://www.ncbi.nlm.nih.gov/pmc/articles/PMC7610201/10.1136/bmj.m3811 PMC7610201 33148618

[9] Guilbert A, Hough I, Seyve E, Rolland M, Quentin J, Slama R, et al. Association of prenatal and postnatal exposures to warm or cold air temperatures with lung function in young infants. JAMA Netw Open. 2023;6:e233376. 36930155 10.1001/jamanetworkopen.2023.3376 PMC10024202

[10] Fang J, Song J, Wu R, Xie Y, Xu X, Zeng Y et al. Association between ambient temperature and childhood respiratory hospital visits in Beijing, China: a time-series study (2013–2017). Environ Sci Pollut Res. 2021 [cited 2024 Apr 28];28:29445–54. Available from: https://link.springer.com/10.1007/s11356-021-12817-w 10.1007/s11356-021-12817-w 33555475

[11] Uibel D, Sharma R, Piontkowski D, Sheffield PE, Clougherty JE. Association of ambient extreme heat with pediatric morbidity: a scoping review. Int J Biometeorol. 2022 [cited 2024 Apr 29];66:1683–98. Available from: https://link.springer.com/10.1007/s00484-022-02310-5 10.1007/s00484-022-02310-5 PMC10019589 35751701

[12] Edwards MJ, Saunders RD, Shiota K. Effects of heat on embryos and foetuses. International Journal of Hyperthermia. 2003 [cited 2024 May 21];19:295–324. Available from: https://www.tandfonline.com/doi/abs/10.1080/0265673021000039628 10.1080/0265673021000039628 12745973

[13] Colson V, Cousture M, Damasceno D, Valotaire C, Nguyen T, Le Cam A, et al. Maternal temperature exposure impairs emotional and cognitive responses and triggers dysregulation of neurodevelopment genes in fish. PeerJ. 2019;7:e6338. 30723624 10.7717/peerj.6338 PMC6360074

[14] Toni M, Angiulli E, Miccoli G, Cioni C, Alleva E, Frabetti F et al. Environmental temperature variation affects brain protein expression and cognitive abilities in adult zebrafish (Danio rerio): A proteomic and behavioural study. Journal of Proteomics. 2019 [cited 2024 Jan 24];204:103396. Available from: https://www.sciencedirect.com/science/article/pii/S187439191930168X 10.1016/j.jprot.2019.103396 31150779

[15] van Wettere WHEJ, Kind KL, Gatford KL, Swinbourne AM, Leu ST, Hayman PT et al. Review of the impact of heat stress on reproductive performance of sheep. Journal of Animal Science and Biotechnology. 2021 [cited 2024 May 21];12:26. Available from: 10.1186/s40104-020-00537-z 10.1186/s40104-020-00537-z PMC7883430 33583422

[16] Wells JCK. Thermal Environment and Human Birth Weight. Journal of Theoretical Biology. 2002 [cited 2024 May 21];214:413–25. Available from: https://www.sciencedirect.com/science/article/pii/S0022519301924658 10.1006/jtbi.2001.2465 11846599

[17] Lian S, Guo J, Wang L, Li W, Wang J, Ji H et al. Impact of prenatal cold stress on placental physiology, inflammatory response, and apoptosis in rats. Oncotarget. 2017 [cited 2024 Apr 16];8:115304–14. Available from: https://www.ncbi.nlm.nih.gov/pmc/articles/PMC5777773/10.18632/oncotarget.23257 PMC5777773 29383161

[18] Bronson SL, Bale TL. The placenta as a mediator of stress effects on neurodevelopmental reprogramming. Neuropsychopharmacology. 2016;41:207–18. 26250599 10.1038/npp.2015.231 PMC4677129

[19] Mao Y, Gao Q, Zhang Y, Yue Y, Ruan T, Yang Y et al. Associations between extreme temperature exposure and hypertensive disorders in pregnancy: a systematic review and meta-analysis. Hypertension in Pregnancy. 2023 [cited 2024 May 22];42:2288586. Available from: https://www.tandfonline.com/doi/full/10.1080/10641955.2023.2288586 10.1080/10641955.2023.2288586 38053322

[20] Kim HY, Cho GJ, Ahn KH, Hong S-C, Oh M-J, Kim H-J. Short-term neonatal and long-term neurodevelopmental outcome of children born term low birth weight. Sci Rep. 2024 [cited 2024 May 21];14:2274. Available from: https://www.nature.com/articles/s41598-024-52154-9 10.1038/s41598-024-52154-9 PMC10821875 38280915

[21] Kong L, Chen X, Liang Y, Forsell Y, Gissler M, Lavebratt C. Association of Preeclampsia and Perinatal Complications With Offspring Neurodevelopmental and Psychiatric Disorders. JAMA Network Open. 2022 [cited 2024 May 21];5:e2145719. Available from: 10.1001/jamanetworkopen.2021.45719 10.1001/jamanetworkopen.2021.45719 PMC8800079 35089349

[22] Puthota J, Alatorre A, Walsh S, Clemente JC, Malaspina D, Spicer J. Prenatal ambient temperature and risk for schizophrenia. Schizophrenia Research. 2022 [cited 2023 Jun 19];247:67–83. Available from: https://www.sciencedirect.com/science/article/pii/S092099642100390X 10.1016/j.schres.2021.09.020 PMC8977400 34620533

[23] Cho H. The effects of summer heat on academic achievement: A cohort analysis. Journal of Environmental Economics and Management. 2017 [cited 2024 May 22];83:185–96. Available from: https://econpapers.repec.org/article/eeejeeman/v_3a83_3ay_3a2017_3ai_3ac_3ap_3a185-196.htm

[24] Duchoslav J. Prenatal Temperature Shocks Reduce Cooperation: Evidence from Public Goods Games in Uganda. Front Behav Neurosci. 2017 [cited 2024 Apr 29];11:249. Available from: https://www.ncbi.nlm.nih.gov/pmc/articles/PMC5742612/10.3389/fnbeh.2017.00249 PMC5742612 29311866

[25] Isen A, Rossin-Slater M, Walker R. Relationship between season of birth, temperature exposure, and later life wellbeing. Proceedings of the National Academy of Sciences. 2017 [cited 2024 Apr 29];114:13447–52. Available from: https://www.pnas.org/doi/10.1073/pnas.1702436114 10.1073/pnas.1702436114 PMC5754756 29203654

[26] Adhvaryu A, Fenske J, Kala N, Nyshadham A. Fetal Origins of Mental Health: Evidence from Africa. Economic Development and Cultural Change. 2024 [cited 2024 Apr 29];72:493–515. Available from: https://www.journals.uchicago.edu/doi/abs/10.1086/722536

[27] Delaney S, Spangler K, Leung M, Nethery R, Tiemeier H, Weisskopf M. Childhood cognitive and behavioral effects of high summer temperatures. ISEE Conference Abstracts. 2022 [cited 2024 May 22];2022. Available from: https://ehp.niehs.nih.gov/doi/abs/10.1289/isee.2022.P-0574

[28] Yeganeh AJ, Reichard G, McCoy AP, Bulbul T, Jazizadeh F. Correlation of ambient air temperature and cognitive performance: A systematic review and meta-analysis. Building and Environment. 2018 [cited 2024 Jan 24];143:701–16. Available from: https://www.sciencedirect.com/science/article/pii/S0360132318304104

[29] Sugg MM, Dixon PG, Runkle JD. Crisis support-seeking behavior and temperature in the united States: is there an association in young adults and adolescents? Sci Total Environ. 2019;669:400–11. 30884264 10.1016/j.scitotenv.2019.02.434

[30] Niu L, Girma B, Liu B, Schinasi LH, Clougherty JE, Sheffield P. Temperature and mental health-related emergency department and hospital encounters among children, adolescents and young adults. Epidemiol Psychiatr Sci. 2023;32:e22. 37066768 10.1017/S2045796023000161 PMC10130844

[31] Essers E, Kusters M, Granés L, Ballester J, Petricola S, Lertxundi N et al. Temperature Exposure and Psychiatric Symptoms in Adolescents From 2 European Birth Cohorts. JAMA Network Open. 2025 [cited 2025 Feb 15];8:e2456898. Available from: 10.1001/jamanetworkopen.2024.56898 10.1001/jamanetworkopen.2024.56898 PMC11775747 39874035

[32] Younan D, Li L, Tuvblad C, Wu J, Lurmann F, Franklin M, et al. Long-Term ambient temperature and externalizing behaviors in adolescents. Am J Epidemiol. 2018;187:1931–41. 29788079 10.1093/aje/kwy104 PMC6118062

[33] Bleses D, Makransky G, Dale PS, Højen A, Ari BA. Early productive vocabulary predicts academic achievement 10 years later. Applied Psycholinguistics. 2016 [cited 2024 May 22];37:1461–76. Available from: https://www.cambridge.org/core/journals/applied-psycholinguistics/article/abs/early-productive-vocabulary-predicts-academic-achievement-10-years-later/7920854715472FBA2FDEB61A6EC21FC8

[34] McGregor KK. How We Fail Children With Developmental Language Disorder. Language, Speech, and Hearing Services in Schools. 2020 [cited 2024 Aug 26];51:981–92. Available from: 10.1044/2020_LSHSS-20-00003 10.1044/2020_LSHSS-20-00003 PMC7842848 32755505

[35] Beitchman JH, Wilson B, Johnson CJ, Atkinson L, Young A, Adlaf E et al. Fourteen-Year Follow-up of Speech/Language-Impaired and Control Children: Psychiatric Outcome. Journal of the American Academy of Child & Adolescent Psychiatry. 2001 [cited 2024 May 22];40:75–82. Available from: https://www.jaacap.org/article/S0890-8567(09)60818-2/abstract 10.1097/00004583-200101000-00019 11195567

[36] López-Ojeda W, Hurley RA. Sexual Dimorphism in Brain Development: Influence on Affective Disorders. JNP. 2021 [cited 2024 Jan 24];33:A4, 85–9. Available from: https://neuro.psychiatryonline.org/doi/10.1176/appi.neuropsych.20100269 10.1176/appi.neuropsych.20100269 34018811

[37] Pallayova M, Brandeburova A, Tokarova D. Update on Sexual Dimorphism in Brain Structure–Function Interrelationships: A Literature Review. Appl Psychophysiol Biofeedback. 2019 [cited 2024 Jan 24];44:271–84. Available from: 10.1007/s10484-019-09443-1 10.1007/s10484-019-09443-1 31218461

[38] Fouillet A, Rey G, Laurent F, Pavillon G, Bellec S, Guihenneuc-Jouyaux C, et al. Excess mortality related to the August 2003 heat wave in France. Int Arch Occup Environ Health. 2006;80:16–24. 16523319 10.1007/s00420-006-0089-4 PMC1950160

[39] Charles MA, Thierry X, Lanoe J-L, Bois C, Dufourg M-N, Popa R, et al. Cohort profile: the French National cohort of children (ELFE): birth to 5 years. Int J Epidemiol. 2020;49:368–j369. 31747017 10.1093/ije/dyz227 PMC7266552

[40] Hough I, Just AC, Zhou B, Dorman M, Lepeule J, Kloog I. A multi-resolution air temperature model for France from MODIS and Landsat thermal data. Environ Res. 2020;183:109244. 32097815 10.1016/j.envres.2020.109244 PMC7167357

[41] Kern S, Langue J, Zesiger P, Bovet F. Adaptations françaises des versions courtes des inventaires du développement communicatif de MacArthur-Bates. Approche Neuropsychologique des Apprentissages chez l’Enfant. 2010 [cited 2024 May 22];107:217–28. Available from: https://www.academia.edu/download/50843183/Adaptations_franaises_des_versions_court20161212-28095-1c26b7q.pdf

[42] Gasparrini A. Distributed lag linear and non-linear models in R: the package dlnm. Journal of statistical software. 2011 [cited 2024 May 22];43:1. Available from: https://www.ncbi.nlm.nih.gov/pmc/articles/PMC3191524/PMC3191524 22003319

[43] Zeka A, Zanobetti A, Schwartz J. Individual-Level Modifiers of the Effects of Particulate Matter on Daily Mortality. American Journal of Epidemiology. 2006 [cited 2024 Jun 30];163:849–59. Available from: 10.1093/aje/kwj116 10.1093/aje/kwj116 16554348

[44] Knol MJ, Pestman WR, Grobbee DE. The (mis)use of overlap of confidence intervals to assess effect modification. Eur J Epidemiol. 2011 [cited 2024 Jul 1];26:253–4. Available from: 10.1007/s10654-011-9563-8 10.1007/s10654-011-9563-8 PMC3088813 21424218

[45] Castagna A, Mascheroni E, Fustinoni S, Montirosso R. Air pollution and neurodevelopmental skills in preschool- and school-aged children: A systematic review. Neuroscience & Biobehavioral Reviews. 2022 [cited 2024 Jan 23];136:104623. Available from: https://www.sciencedirect.com/science/article/pii/S0149763422001129 10.1016/j.neubiorev.2022.104623 35331818

[46] Liew Z, Olsen J, Cui X, Ritz B, Arah OA. Bias from conditioning on live birth in pregnancy cohorts: an illustration based on neurodevelopment in children after prenatal exposure to organic pollutants. Int J Epidemiol. 2015;44:345–54. 25604449 10.1093/ije/dyu249 PMC4339763

[47] Wilde J, Apouey B, Jung T. The effect of ambient temperature shocks during conception and early pregnancy on later life outcomes. Eur Econ Rev. 2017;97:87–107.

[48] Guo T, Wang Y, Zhang H, Zhang Y, Zhao J, Wang Y, et al. The association between ambient temperature and the risk of preterm birth in China. Sci Total Environ. 2018;613–614:439–46. 10.1016/j.scitotenv.2017.09.104 28918275

[49] Cox B, Vicedo-Cabrera A, Gasparrini A, Roels H, Martens E, Vangronsveld J et al. Ambient temperature as a trigger of preterm delivery in a temperate climate. J Epidemiol Commun Health. 2016;70:1191–99. 10.1136/jech-2015-206384 10.1136/jech-2015-206384 27261529

[50] Granés L, Essers E, Ballester J, Petricola S, Tiemeier H, Iñiguez C et al. Early life cold and heat exposure impacts white matter development in children. Nat Clim Chang. 2024 [cited 2024 Aug 20];14:760–6. Available from: https://www.nature.com/articles/s41558-024-02027-w

[51] Lebel C, Deoni S. The Development of Brain White Matter Microstructure. Neuroimage. 2018 [cited 2024 May 22];182:207–18. Available from: https://www.ncbi.nlm.nih.gov/pmc/articles/PMC6030512/10.1016/j.neuroimage.2017.12.097 PMC6030512 29305910

[52] Dubois J, Dehaene-Lambertz G, Kulikova S, Poupon C, Hüppi PS, Hertz-Pannier L. The early development of brain white matter: a review of imaging studies in fetuses, newborns and infants. Neuroscience. 2014;276:48–71. 24378955 10.1016/j.neuroscience.2013.12.044

[53] Murage P, Hajat S, Kovats RS. Effect of night-time temperatures on cause and age-specific mortality in London. Environ Epidemiol. 2017 [cited 2024 May 23];1:e005. Available from: https://www.ncbi.nlm.nih.gov/pmc/articles/PMC7608908/10.1097/EE9.0000000000000005 PMC7608908 33195962

[54] Laaidi K, Zeghnoun A, Dousset B, Bretin P, Vandentorren S, Giraudet E et al. The Impact of Heat Islands on Mortality in Paris during the August 2003 Heat Wave. Environ Health Perspect. 2012 [cited 2024 Aug 26];120:254–9. Available from: https://www.ncbi.nlm.nih.gov/pmc/articles/PMC3279432/10.1289/ehp.1103532 PMC3279432 21885383

[55] Nelson ca. From Neurons to Neighborhoods: The Science of Early Childhood Development. Shonkoff JP, Phillips DA, editors. Washington (DC): National Academies Press (US); 2000 [cited 2024 Aug 26]. Available from: http://www.ncbi.nlm.nih.gov/books/NBK225557/25077268

[56] Adebiyi OE, Adigun KO, Adebiyi AI, Odenibi BS. High environmental temperature: insights into behavioural, neurodevelopmental and gut Microbiome changes following gestational exposure in rats. Neuroscience. 2022;488:60–76. 35231581 10.1016/j.neuroscience.2022.02.026

[57] Eriksson JG, Kajantie E, Osmond C, Thornburg K, Barker DJP. Boys live dangerously in the womb. American J Hum Biol. 2010 [cited 2024 May 22];22:330–5. Available from: https://onlinelibrary.wiley.com/doi/10.1002/ajhb.20995 10.1002/ajhb.20995 PMC3923652 19844898

[58] Mondal D, Galloway TS, Bailey TC, Mathews F. Elevated risk of stillbirth in males: systematic review and meta-analysis of more than 30 million births. BMC Medicine. 2014 [cited 2024 May 22];12:220. Available from: 10.1186/s12916-014-0220-4 10.1186/s12916-014-0220-4 PMC4245790 25428603

[59] Randazzo T, De Cian E, Mistry MN. Air conditioning and electricity expenditure: The role of climate in temperate countries. Economic Modelling. 2020 [cited 2024 Aug 20];90:273–87. Available from: https://ideas.repec.org//a/eee/ecmode/v90y2020icp273-287.html

