# Supplementary material for: Impact of early life exposure to heat and cold on linguistic development in two-year-old children: findings from the ELFE cohort study
Source: Environ Health. 2025 Apr 9;24:19. doi: 10.1186/s12940-025-01173-8 (PMC11980264; doi:10.1186/s12940-025-01173-8)
Supplement: Supplementary file 1 — Supplementary Material 1 [file 12940_2025_1173_MOESM1_ESM.docx]

**Supplementary Materials**

**Impact of early life exposure to heat and cold on linguistic development in two-year-old children. Findings from the ELFE cohort study.**

Table of Contents

[Supplementary Method. Description of the covariates*.* 2](#__RefHeading___Toc34056_2138783787)

[Supplementary Table 1. Comparison of the imputed and not imputed datasets. 5](#__RefHeading___Toc34058_2138783787)

[Supplementary Table 2. Association of prenatal and postnatal cumulative exposure to heat and cold with the MB-CDI score from models unadjusted for parental behaviors (and unadjusted for pollution). 9](#__RefHeading___Toc34062_2138783787)

[Legend. MB-CDI, MacArthur-Bates Communicative Development Inventories; pct, percentile; Wk, Week. 9](#__RefHeading___Toc34060_2138783787)

[Supplementary Table 3. Association of prenatal and postnatal cumulative exposure to severe heat and cold with the MB-CDI score (from models adjusted for pollution). 10](#__RefHeading___Toc14648_646822602)

[Supplementary Table 4. Association of prenatal and postnatal cumulative exposure to heat and cold with the MB-CDI score, stratified by sex (from models unadjusted for pollution). 12](#__RefHeading___Toc34064_2138783787)

[Supplementary Figure 1. Directed acyclic graph. 14](#__RefHeading___Toc34066_2138783787)

[Supplementary Figure 2. Distribution of weekly overall temperatures during the study period. 16](#__RefHeading___Toc34068_2138783787)

[Supplementary Figure 3. Distribution of the MB-CDI score. 17](#__RefHeading___Toc34070_2138783787)

[Supplementary Figure 4. Lag-specific effect of Heat on the MB-CDI score (from models adjusted for PM2.5). 18](#__RefHeading___Toc34072_2138783787)

[Supplementary Figure 5. Lag-specific effect of Heat on the MB-CDI score (from models adjusted for PM10). 19](#__RefHeading___Toc34074_2138783787)

[Supplementary Figure 6. Lag-specific effect of Heat on the MB-CDI score (from models adjusted for NO2). 20](#__RefHeading___Toc34076_2138783787)

[Supplementary Figure 7. Lag-specific effect of Cold on the MB-CDI score (from models adjusted for PM2.5). 21](#__RefHeading___Toc34078_2138783787)

[Supplementary Figure 8. Lag-specific effect of Cold on the MB-CDI score (from models adjusted for PM10). 22](#__RefHeading___Toc34080_2138783787)

[Supplementary Figure 9. Lag-specific effect of Cold on the MB-CDI score (from models adjusted for NO2). 23](#__RefHeading___Toc34082_2138783787)

[Supplementary Figure 10. Lag-specific effects of Heat on the MB-CDI score in infant boys and girls (from models unadjusted for pollution). 24](#__RefHeading___Toc34084_2138783787)

[Supplementary Figure 11. Lag-specific effects of Cold on the MB-CDI score in infant boys and girls (from models unadjusted for pollution). 25](#__RefHeading___Toc34086_2138783787)

[References. 26](#__RefHeading___Toc34088_2138783787)

# **Supplementary Method. Description of the covariates*.***

Covariates were identified based on literature findings which were further translated in a directed acyclic graph (**Supplementary Figure 1A**).

- Parents: socio-economic status and demographic indicators.

Numerous studies have demonstrated that a high socio-economic status – characterized by factors such as high household income, maternal education, and professional occupation –can create a supportive environment for language development [1]. Parental age at conception has also been related to a child’s cognitive abilities: on the one hand, high maternal [2] and paternal age [3,4] at conception have been related to a range of adverse perinatal outcomes. Yet on the other hand, advanced maternal age has been related to a socioeconomically advantaged family background [5]. Children also exhibit lower performances in language abilities when their parents are separated [6], though this finding is controversial [7]. Finally, others have reported cognitive development disparities based on racial or ethnic origins [8].

Consequently, we adjusted our analyses on parental education, measured when the child was two months of age (highest level among mother and father: primary or intermediary school vs. high-school vs. undergraduate diploma (Baccalaureate +two years) vs. graduate diploma (Baccalaureate +two to five years) vs. postgraduate diploma (Baccalaureate +five years or more)); maternal and paternal age at conception (categorical – five year bins); socio-economic position of the mother, measured in the second trimester (executives and higher intellectual professions vs. intermediate professions vs. employees vs. workers vs. craftsmen or merchants vs. others); household income, measured when the child was two years of age (linearly ordered quintiles); and whether parents were living together when the child was two years of age (no vs. yes). Though we did not record race and ethnicity because racial statistics are not allowed in France [9], we adjusted our analysis on the mother’s birthplace (France vs. other).

*- Neighborhood: socio-economic context and urbanization.*

Both socially deprived and urbanized areas may show higher levels of temperature [10,11]. On the other hand, a child’s neighbourhood, and especially its socio-cultural context, may influence the quality of his/her home learning environment [12], and in turn decrease his/her learning abilities.

We adjusted our analyses for an index of urbanization, measured when the child was one year of age, with three different categories based on the size of the urban area (not an urban area or urban area with less than 50,000 inhabitants vs. urban area with 50,000 to 500,000 inhabitants vs. urban area with more than 500,000 inhabitants). We also adjusted for the European Deprivation Index obtained for the year 2011, at the smallest geographical unit available in France, which is the census block group level, known as IRIS in Metropolitan France (EDI [13]). The EDI was categorized in three different groups based on tertiles to account for non-linear effects.

*- Neighbourhood: vegetation.*

Vegetation can affect thermal conditions [14] and cognitive development [15,16]. We adjusted for the normalized difference vegetation index (NDVI) as the mean Landsat satellite NDVI in a 100 m buffer around home address during the months of June, July and August of years 2011, 2012, 2013. Values calculated with less than 75% observations were removed and considered missing. NDVI was further categorized in three different groups based on tertiles to account for non-linear effects.

*- Languages spoken at home.*

Language exposure has been shown to have an effect on language development [17]. The number of languages spoken at home, measured when the child was two years of age, was included as a covariate in our analysis (one vs. two vs. three or more).

*- Pre-pregnancy history: parity.*

It has been argued that first-born children have better cognitive outcomes than later-born children, potentially because they have greater access to parental resources [18]. Also, there may be a negative relationship between the size of the family and the level of household income [5,19], which may explain why large family size is negatively associated with early cognitive development [20]. Parity (primiparous vs. multiparous) was included as a covariate.

*- Pre-pregnancy history: neurodevelopmental difficulties.*

A parental history of neurodevelopmental issues may predispose to neurodevelopmental disorders in the offspring. Consequently, we adjusted our analysis for mother’s and father’s history of problems with mathematics, reading, writing, communication and language (at least one issue vs. no impairment).

*- Pre-pregnancy history: maternal obesity.*

Maternal obesity has been shown to be associated with neurodevelopment of two-year-old children [21]. We therefore adjusted for maternal Body Mass Index (BMI) before pregnancy (BMI<18.5 vs. BMI between 18.5 and 25 vs. BMI between 25 and 30 vs. BMI>30).

*- Food and drug exposure during pregnancy and after birth.*

Maternal supplementation in vitamin B9 is thought to facilitate brain development [22]. Smoking during pregnancy has been suggested to reduce speech processing abilities [23], see however contradictory findings [24], and [25] for review). Studies have demonstrated a negative effect of alcohol consumption during pregnancy on children’s language abilities [26]. Caffeine exposure has been negatively related to children’s full-scale and performance IQ (but not verbal IQ) compared with low caffeine exposure [27]. Yet, no significant associations were found between caffeine intake during pregnancy and language difficulties at the age of 18 months [28]. Maternal fish consumption has been associated with a child’s language abilities at age three [29,30]. Fish is thought to be beneficial to the fetus via their content in poly-unsaturated fatty acid (a.k.a. omega 3). For instance, a recent study has found that mothers consuming omega-3 in the second half of pregnancy were 40% less likely to have children with autism spectrum disorders [31]. Breastfeeding has been associated with a range of positive neurodevelopmental outcomes [32].

We adjusted our analysis for maternal supplementation in vitamin B9, measured in the third trimester (no vs. yes); smoking during pregnancy, measured in the second trimester (not exposed vs. actively or passively exposed); alcohol use during pregnancy, measured in the second trimester (less than once per month vs. more than once per month); coffee consumption during pregnancy, measured in the third trimester (less than once a day vs. more than once a day); fish consumption during pregnancy, measured in the second trimester (never vs. less than once a month vs. once to three times a month vs. once a week vs. more than once a week; linearly ordered); fatty acid supplementation, measured in the third trimester (no vs. yes); and breastfeeding, measured when the child was two months of age (breastfeeding only vs. breastfeeding and bottle feeding vs. bottle feeding only).

Finally, while we included certain covariates related to food and drug exposure in our main analysis, it is important to acknowledge that previous studies have suggested that climatic conditions may influence breastfeeding practices [33], as well as alcohol [34] and tobacco consumption [35]. Consequently, pre- and post-natal food/drug exposure could potentially lie in the causal pathway between the exposure and outcome of interest (**Supplementary Figure 1B**). We conducted a sensitivity analysis "neglecting" pre- and post-natal food/drug exposure to take into account the potential mediating role of these factors.

*- Age and sex of the child.*

Boys have been shown to be a bit delayed in their language development [36]. We included the sex of the child (male vs. female) as a covariate in the analysis and subsequently stratified our analysis by sex (see below). We also included the child's exact age (in months) at the time the MB-CDI was scored as a covariate.

*- Air pollution.*

Ambient temperature has been suggested to influence air pollution concentration via its effect on emissions, atmospheric chemistry and pollutant transport [37,38]. In our baseline analysis, we investigated the total effect of temperature on language development, not accounting for pollution. In a series of secondary analyses, we took into account atmospheric pollutants (PM2.5, PM10, NO2) in our statistical model to estimate the effect of temperature after ambient pollution has been explained away.

We estimated daily ambient pollution at mother’s and child’s home address using highly resolved spatiotemporal modeling. For each pollutant, our geospatial model used a multi-stage ensemble approach combining three basis learners (among which: linear mixed models, random forests, gradient boosting, categorical boosting, and gaussian markov random field) to calibrate concentrations measured at monitoring stations with spatiotemporal predictors. For PM2.5 and PM10, we used predictions from a model that estimated daily PM2.5 and PM10 concentrations from 2000 to 2019 at a 1 km spatial resolution across France. Models performed with an overall cross-validated R^2^ of 0.76 for PM2.5 and of 0.71 for PM10 [39]. NO2 predictions were estimated from 2005 to 2022 at a 1 km spatial resolution across France and at a 200 m spatial resolution over urban areas with >50,000 inhabitants [40] . Models performed with cross-validated R^2^ for the 1km predictions of 0.83 and for the 200 m predictions of 0.69.

*- Pregnancy, birth issues and medical history of the child.*

Though potentially involved in neurodevelopment, we did not adjust for pregnancy and birth outcomes, as well as medical issues during infancy. Indeed, the latter may be caused by ambient temperatures that are above or below average and may be considered mediators.

*- Factors that may be influenced by linguistic development.*

External stimulation (whether from parents, other adults or children), childcare and preschool attendance, can influence children’s ability to speak – e.g. via language input [41]. Association between screen use and language development has also proven significant, with greater quantity of screen use being negatively associated with child language [42]. Sleep is another feature typically involved in language development [43].

The reverse however may also be true. Preschool children with language impairment have been shown to suffer from emotional (e.g. understanding emotion meaning [44]) and behavioral problems (e.g. hyperactivity [45]), as well as poor social competence [46], and have been found to be more dependent and isolated [47]. The latter in turn may influence the quality and quantity of external stimulations, childcare or preschool attendance, and quality of sleep [48,49].

In addition, evidence of bidirectional associations between sensitive parenting and language skills has been observed, especially in boys. For instance, boys' receptive language skills at 24 months uniquely contributed to increased sensitive parenting by mothers from 24 to 36 months [50]. Another study reported evidence of child-to-parent effects, where children with higher language skills tend to elicit more complex language input from their parents [51].

Accordingly, we reasoned that language development may influence stimulation and activities, childcare, preschool attendance, screen use and sleep, and that adjusting our analysis for these factors would risk reverse causation bias.

*- Birth seasonality.*

We did not include birth seasonality in our set of covariates because we found no evidence in the literature suggesting that birth seasonality may affect neurodevelopment independently of its association with temperature. We therefore concluded that birth seasonality is unlikely to confound the relationship between temperature and neurodevelopment. Adjusting for seasonality could potentially remove a substantial part of the temperature variation, leading to over-adjustment and biased results [52].

# **Supplementary Table 1. Comparison of the imputed and not imputed datasets.**

| **Variables** | Not Imputed (N=12163) | Imputed (N=12163) | P-value |
| --- | --- | --- | --- |
| **European Defavor Index of living area** |  |  |  |
| Low: (-9.18,-1.6] | 4194 (34.5%) | 4203 (34.6%) | 1 |
| Medium: (-1.6,2.06] | 4434 (36.5%) | 4437 (36.5%) |  |
| High: (2.06,31.9] | 3519 (28.9%) | 3523 (29.0%) |  |
| Missing | 16 (0.1%) | 0 (0%) |  |
| **NDVI^a^** |  |  |  |
| Low: (0.0367,0.393] | 3732 (30.7%) | 3733 (30.7%) | 1 |
| Medium: (0.393,0.51] | 4066 (33.4%) | 4066 (33.4%) |  |
| High: (0.51,0.89] | 4362 (35.9%) | 4364 (35.9%) |  |
| Missing | 3 (0.0%) | 0 (0%) |  |
| **Size of living area** |  |  |  |
| Rural area or less than 50,000 inhabitants | 2447 (20.1%) | 2519 (20.7%) | 0.97 |
| 50,000 to 500,000 inhabitants | 3778 (31.1%) | 3922 (32.2%) |  |
| More than 500,000 inhabitants | 5543 (45.6%) | 5722 (47.0%) |  |
| Missing | 395 (3.2%) | 0 (0%) |  |
| **Mother’s birth place** |  |  |  |
| France | 10903 (89.6%) | 10973 (90.2%) | 0.98 |
| Other | 1185 (9.7%) | 1190 (9.8%) |  |
| Missing | 75 (0.6%) | 0 (0%) |  |
| **Mother’s history of learning difficulties** |  |  |  |
| No | 6715 (55.2%) | 6944 (57.1%) | 0.61 |
| Yes | 4978 (40.9%) | 5219 (42.9%) |  |
| Missing | 470 (3.9%) | 0 (0%) |  |
| **Father’s history of learning difficulties** |  |  |  |
| No | 4964 (40.8%) | 5914 (48.6%) | 0.1 |
| Yes | 5017 (41.2%) | 6249 (51.4%) |  |
| Missing | 2182 (17.9%) | 0 (0%) |  |
| **Level of education (Highest between mother and father)** |  |  |  |
| Primary or secondary school | 264 (2.2%) | 278 (2.3%) | 0.98 |
| Highschool | 2931 (24.1%) | 3004 (24.7%) |  |
| Undergraduate | 2675 (22.0%) | 2714 (22.3%) |  |
| Bachelor | 2191 (18.0%) | 2219 (18.2%) |  |
| Postgraduate | 3914 (32.2%) | 3948 (32.5%) |  |
| Missing | 188 (1.5%) | 0 (0%) |  |
| **Mother’s socio-professional category** |  |  |  |
| Craftsmen & merchants | 1010 (8.3%) | 1033 (8.5%) | 0.98 |
| executives and higher intellectual professions | 3011 (24.8%) | 3056 (25.1%) |  |
| intermediate professions | 1739 (14.3%) | 1765 (14.5%) |  |
| Employees (skilled and unskilled) | 4234 (34.8%) | 4318 (35.5%) |  |
| Workers (skilled and unskilled) | 1377 (11.3%) | 1424 (11.7%) |  |
| Others | 532 (4.4%) | 567 (4.7%) |  |
| Missing | 260 (2.1%) | 0 (0%) |  |
| **Parity** |  |  |  |
| Primiparous | 5531 (45.5%) | 5556 (45.7%) | 0.97 |
| Multiparous | 6586 (54.1%) | 6607 (54.3%) |  |
| Missing | 46 (0.4%) | 0 (0%) |  |
| **Pre-pregnancy Body Mass Index** |  |  |  |
| <18.5 | 868 (7.1%) | 875 (7.2%) | 1 |
| 18.5-25 | 8053 (66.2%) | 8134 (66.9%) |  |
| 25-30 | 2010 (16.5%) | 2025 (16.6%) |  |
| >30 | 1120 (9.2%) | 1129 (9.3%) |  |
| Missing | 112 (0.9%) | 0 (0%) |  |
| **Mother’s age at conception** |  |  |  |
| 25 or below | 1139 (9.4%) | 1141 (9.4%) | 1 |
| 26-30 | 3988 (32.8%) | 3997 (32.9%) |  |
| 31-35 | 4521 (37.2%) | 4524 (37.2%) |  |
| 36-40 | 2046 (16.8%) | 2048 (16.8%) |  |
| 41 or above | 453 (3.7%) | 453 (3.7%) |  |
| Missing | 16 (0.1%) | 0 (0%) |  |
| **Father’s age at conception** |  |  |  |
| 25 or below | 554 (4.6%) | 580 (4.8%) | 1 |
| 26-30 | 2730 (22.4%) | 2807 (23.1%) |  |
| 31-35 | 4332 (35.6%) | 4447 (36.6%) |  |
| 36-40 | 2693 (22.1%) | 2766 (22.7%) |  |
| 41-45 | 1048 (8.6%) | 1086 (8.9%) |  |
| 46 or above | 463 (3.8%) | 477 (3.9%) |  |
| Missing | 343 (2.8%) | 0 (0%) |  |
| **Alcohol consumption during pregnancy** |  |  |  |
| At least once a month | 3128 (25.7%) | 3146 (25.9%) | 0.98 |
| Never or less than once a month | 8955 (73.6%) | 9017 (74.1%) |  |
| Missing | 80 (0.7%) | 0 (0%) |  |
| **Consumption of tobacco during pregnancy** |  |  |  |
| At least some exposure (passive or active) | 3844 (31.6%) | 3930 (32.3%) | 0.71 |
| No exposure | 7968 (65.5%) | 8233 (67.7%) |  |
| Missing | 351 (2.9%) | 0 (0%) |  |
| **Coffee consumption during pregnancy** |  |  |  |
| Less than once a day | 9966 (81.9%) | 11044 (90.8%) | 0.68 |
| Once a day or more | 1030 (8.5%) | 1119 (9.2%) |  |
| Missing | 1167 (9.6%) | 0 (0%) |  |
| **Fish consumption during pregnancy** |  |  |  |
| Never | 613 (5.0%) | 672 (5.5%) | 1 |
| Less than once a month | 1313 (10.8%) | 1446 (11.9%) |  |
| One to three times a month | 3294 (27.1%) | 3615 (29.7%) |  |
| Once a week | 3714 (30.5%) | 4047 (33.3%) |  |
| Twice a week or more | 2160 (17.8%) | 2383 (19.6%) |  |
| Missing | 1069 (8.8%) | 0 (0%) |  |
| **Vitamin B9 consumption during pregnancy** |  |  |  |
| No | 5250 (43.2%) | 5395 (44.4%) | 0.79 |
| Yes | 6634 (54.5%) | 6768 (55.6%) |  |
| Missing | 279 (2.3%) | 0 (0%) |  |
| **Omega 3 consumption during pregnancy** |  |  |  |
| Less than once a week | 2567 (21.1%) | 3089 (25.4%) | 0.98 |
| More than once a week | 1796 (14.8%) | 2147 (17.7%) |  |
| Never | 5792 (47.6%) | 6927 (57.0%) |  |
| Missing | 2008 (16.5%) | 0 (0%) |  |
| **Sex of the child** |  |  |  |
| Female | 5988 (49.2%) | 5988 (49.2%) | 1 |
| Male | 6175 (50.8%) | 6175 (50.8%) |  |
| **Feeding method at 2 months** |  |  |  |
| Breastfeeing only | 3963 (32.6%) | 4058 (33.4%) | 0.99 |
| Breastfeeing & Bottle feeding | 1927 (15.8%) | 1966 (16.2%) |  |
| Bottle feeding only | 5987 (49.2%) | 6139 (50.5%) |  |
| Missing | 286 (2.4%) | 0 (0%) |  |
| **Parental relationship** |  |  |  |
| Separated | 690 (5.7%) | 701 (5.8%) | 0.91 |
| Together | 11372 (93.5%) | 11462 (94.2%) |  |
| Missing | 101 (0.8%) | 0 (0%) |  |
| **Number of languages spoken at home** |  |  |  |
| One | 8566 (70.4%) | 8711 (71.6%) | 0.93 |
| Two | 2525 (20.8%) | 2595 (21.3%) |  |
| Three or more | 834 (6.9%) | 857 (7.0%) |  |
| Missing | 238 (2.0%) | 0 (0%) |  |
| **Household income** |  |  |  |
| 1^st^ quintile | 2168 (17.8%) | 2305 (19.0%) | 1 |
| 2^nd^ quintile | 2349 (19.3%) | 2476 (20.4%) |  |
| 3^rd^ quintile | 2286 (18.8%) | 2421 (19.9%) |  |
| 4^th^ quintile | 2353 (19.3%) | 2472 (20.3%) |  |
| 5^th^ quintile | 2365 (19.4%) | 2489 (20.5%) |  |
| Missing | 642 (5.3%) | 0 (0%) |  |
| **Age at MB-CDI test (month)** |  |  |  |
| Mean [min, max] | 25.33 [23.00, 28.00] | 25.33 [23.00, 28.00] | 0.79 |
| Missing | 101 (0.8%) | 0 (0%) |  |

Legend. NDVI, Normalized difference vegetation index; MB-CDI, MacArthur-Bates Communicative Development Inventories; Q1, 1^st^ quartile; Q3, 3^rd^ quartile.

# **Supplementary Table 2. Association of prenatal and postnatal cumulative exposure to heat and cold with the MB-CDI score from models unadjusted for parental behaviors (and unadjusted for pollution).**

|  | Prenatal period | | Postnatal period | |
| --- | --- | --- | --- | --- |
| Exposure | Temperature | Cumulative effect | **Temperature** | **Cumulative effect** |
| Severe Overall Heat | Tmean=21.2°C vs. 13.9°C | Wk 1-3: 1.037  (1.001-1.074) | Tmean=21.9°C vs. 11.5°C | Wk 1-27: 0.861  (0.765-0.969) |
| Severe Daytime Heat | Tmax=27.9°C vs. 19.9°C | Wk 1-5: 1.053  (1.004-1.104) | Tmax=28.8°C vs. 16.8°C | Wk 16-27: 0.965  (0.935-0.996) |
| Severe Night-time Heat | Tmin=15.6°C vs. 8.3°C | Wk 13-19: 0.962 (0.929-0.997) | Tmin=16.0°C vs. 7.3°C | Wk 10-24: 0.954  (0.914-0.995) |
| Severe Night-time Cold | Tmin=-1.5°C vs.  8.3°C | Wk 15-22: 1.048 (1.009-1.088) | Tmin=-1.8°C vs. 7.3°C | No critical window |

In this model, we excluded the following covariates from our analysis: fish consumption, coffee and alcohol intake, tobacco use, omega-3 and vitamin B9 supplementation during pregnancy, and breastfeeding practices. Cumulative adjusted risk ratio associated with severe heat (95^th^ percentile vs. 50^th^ percentile) and cold (5^th^ percentile vs. 50^th^ percentile) throughout an entire critical window. 95% confidence intervals are also reported. Risks lower than one indicate that heat or cold, when compared to the median temperature, are associated with a reduction in the MB-CDI score. Conversely, risks higher than one indicate that heat or cold, relative to the median temperature, are linked to an increase in the MB-CDI score.

# Legend. MB-CDI, MacArthur-Bates Communicative Development Inventories; pct, percentile; Wk, Week.

# **Supplementary Table 3. Association of prenatal and postnatal cumulative exposure to severe heat and cold with the MB-CDI score (from models adjusted for pollution).**

| Exposure | Prenatal period  Cumulative effect | Postnatal period  Cumulative effect |
| --- | --- | --- |
| ***Severe Overall Heat*** | *Tmean=21.2 vs. 13.9°C* | *Tmean=21.9 vs. 11.5°C* |
| Adj. on PM2.5 | No critical window | Wk 5-25: 0.902  (0.823-0.989) |
| Adj. on PM10 | No critical window | Wk 1-26: 0.866  (0.765-0.982) |
| Adj. on NO2 | Wk 1: 1.014  (0.999-1.029)  &  Wk 15-20: 0.966  (0.934-0.998) | Wk 1-28: 0.849  (0.751-0.961) |
| Adj. on PM2.5 & NO2 | No critical window | Wk 1-27: 0.855  (0.752-0.973) |
| Adj. on PM10 & NO2 | No critical window | Wk 1-28: 0.848  (0.744-0.966) |
| ***Severe Daytime*** ***Heat*** | *Tmax=27.9 vs. 19.9°C* | *Tmax=28.8 vs. 16.8°C* |
| Adj. on PM2.5 | No critical window | No critical window |
| Adj. on PM10 | No critical window | No critical window |
| Adj. on NO2 | Wk 1-5: 1.055  (1.005-1.108) | Wk 18-27: 0.972  (0.947-0.997) |
| Adj. on PM2.5 & NO2 | Wk 1-5: 1.056  (1.004-1.110) | No critical window |
| Adj. on PM10 & NO2 | Wk 1-5: 1.054  (1.003-1.109) | Wk 21-28: 0.980  ( 0.961-0.998) |
| ***Severe Night-time Heat*** | *Tmin=15.6 vs. 8.3°C* | *Ref: Tmin=16.0 vs. 7.3°C* |
| Adj. on PM2.5 | Wk 1-5: 1.052  (1.005-1.102) | No critical window |
| Adj. on PM10 | Wk 1-3: 1.035  (1.002-1.069)  &  Wk 17-18: 0.990  (0.979-0.999) | No critical window |
| Adj. on NO2 | Wk 1-4: 1.044  (1.003 1.086)  &  Wk 14-20: 0.963  (0.930-0.997) | Wk 14-22: 0.975  (0.951-0.999) |
| Adj. on PM2.5 & NO2 | Wk 1-4: 1.046  (1.004-1.089) | No critical window |
| Adj. on PM10 & NO2 | Wk 1-4: 1.045  (1.003-1.089)  &  Wk 16-19: 0.979  (0.959-0.999) | No critical window |
| ***Severe Night-time Cold*** | *Tmin=-1.5 vs. 8.3°C* | *Tmin=-1.8 vs. 7.3°C* |
| Adj. on PM2.5 | Wk 14-23: 1.073  (1.019-1.129) | Wk 65-70: 0.977  (0.955-0.999) |
| Adj. on PM10 | Wk 14-22: 1.063  (1.014-1.115) | Wk 66-70: 0.981  (0.963-0.999) |
| Adj. on NO2 | Wk 16-22: 1.044  (1.009-1.080) | No critical window |
| Adj. on PM2.5 & NO2 | Wk 14-22: 1.066  (1.015-1.119) | Wk 67-69: 0.989  (0.977-0.999) |
| Adj. on PM10 & NO2 | Wk 14-22: 1.062  (1.013-1.115) | No critical window |

Cumulative adjusted risk ratio associated with severe heat (95^th^ percentile vs. 50^th^ percentile) and cold (5^th^ percentile vs. 50^th^ percentile) throughout an entire critical window. 95% confidence interval are also reported. Risks lower than one indicate that heat or cold, when compared to the median temperature, are associated with a reduction in the MB-CDI score. Conversely, risks higher than one indicate that heat or cold, relative to the median temperature, are linked to an increase in the MB-CDI score.

Legend. MB-CDI, MacArthur-Bates Communicative Development Inventories; Adj., Adjusted; Wk, Week.

# **Supplementary Table 4. Association of prenatal and postnatal cumulative exposure to heat and cold with the MB-CDI score, stratified by sex (from models unadjusted for pollution).**

| Exposure/ Period | Males | Females | Ratio  Males/Females |
| --- | --- | --- | --- |
| ***Overall Heat/ Postnatal period***  ***Ref (50^th^ pct): Tmean=11.5°C*** | | | |
| Moderate (90^th^ pct) Tmean=20.0°C  Wk 1-22 | **0.857**  **(0.747-0.983)** | 0.935  (0.841-1.04) | 0.917  (0.762-1.071) |
| Severe (95^th^ pct) Tmean=21.9°C  Wk 1-21 | **0.821**  **(0.690-0.977)** | 0.923  (0.806-1.055) | 0.889  (0.7-1.079) |
| Extreme (99^th^ pct) Tmean=24.8°C  Wk 1-21 | **0.758**  **(0.594-0.967)** | 0.898  (0.744-1.084) | 0.844  (0.592-1.096) |
| ***Overall Heat/ Postnatal period***  ***Ref (50^th^ pct): Tmean=11.5°C*** | | | |
| Moderate (90^th^ pct) Tmean=20.0°C  Wk 23-27 | 0.989  (0.976-1.003) | **0.989**  **(0.979-0.999)** | 1.000  (0.983-1.017) |
| Severe (95^th^ pct) Tmean=21.9°C  Wk 22-29 | 0.980  (0.953-1.008) | **0.977**  **(0.956-0.998)** | 1.003  (0.968-1.038) |
| Extreme (99^th^ pct) Tmean=24.8°C  Wk 22-31 | 0.971  (0.925-1.020) | **0.958**  **(0.922-0.994)** | 1.014  (0.954-1.073) |
| ***Daytime Heat/ Postnatal period***  ***Ref (50^th^ pct): Tmax=16.8°C*** | | | |
| Moderate (90^th^ pct) Tmax=25.6°C  Wk 21-32 | 0.991  (0.963-1.020) | **0.972**  **(0.951-0.994)** | 1.020  (0.984-1.055) |
| Severe (95^th^ pct) Tmax=28.8°C  Wk 21-33 | 0.989  (0.949 -1.030) | **0.960**  **(0.930-0.990)** | 1.030  (0.980-1.081) |
| Extreme (99^th^ pct) Tmax=32.1°C  Wk 21-34 | 0.986  (0.926-1.051) | **0.938**  **(0.895-0.984)** | 1.051  (0.974-1.128) |
| ***Night-time Cold/ Prenatal period***  ***Ref (50^th^ pct): Tmin=8.3°C*** | | | |
| Moderate (10^th^ pct) Tmin=0.1°C  Wk 15-21 | **1.049**  **(1.004-1.097)** | 1.016  (0.981-1.052) | 1.032  (0.974-1.091) |
| Severe (5^th^ pct) Tmin=-1.5°C  Wk 16-20 | **1.044**  **(1.003-1.087)** | 1.012  (0.980-1.045) | 1.032  (0.979-1.085) |
| Extreme (1^st^ pct) Tmin=-4.0°C  Wk 17-20 | **1.045**  **(1.002-1.09)** | 1.011  (0.978-1.045) | 1.034  (0.978-1.089) |
| ***Night-time Cold/ Postnatal period***  ***Ref (50^th^ pct): Tmin=7.3°C*** | | | |
| Moderate (10^th^ pct) Tmin=0.2°C  Wk 34-64 | 1.008  (0.849-1.197) | **0.864**  **(0.758-0.984)** | 1.167  (0.959-1.374) |
| Severe (5^th^ pct) Tmin=-1.8°C  Wk 36-67 | 1.007  (0.786-1.29) | **0.809**  **(0.671-0.976)** | 1.245  (0.950-1.539) |
| Extreme (1^st^ pct) Tmin=-7.3°C  Wk 37-67 | 1.031  (0.637-1.668) | **0.668**  **(0.463-0.962)** | 1.543  (0.971-2.116) |

Critical windows and cumulative adjusted risk ratios associated with ambient temperature exposure were identified in males and females separately. 95% confidence interval are also reported. Significant risk ratios are in bold. Risks lower than one indicate that heat or cold, when compared to the median temperature, are associated with a reduction in the MB-CDI score. Conversely, risks higher than one indicate that heat or cold, relative to the median temperature, are linked to an increase in the MB-CDI score.

Ratios of risk ratio are also reported but none of the between-group differences were significant.

Legend. MB-CDI, MacArthur-Bates Communicative Development Inventories; pct, percentile; Wk, Week.

# Supplementary Figure 1. Directed acyclic graph.

**A. Main model**


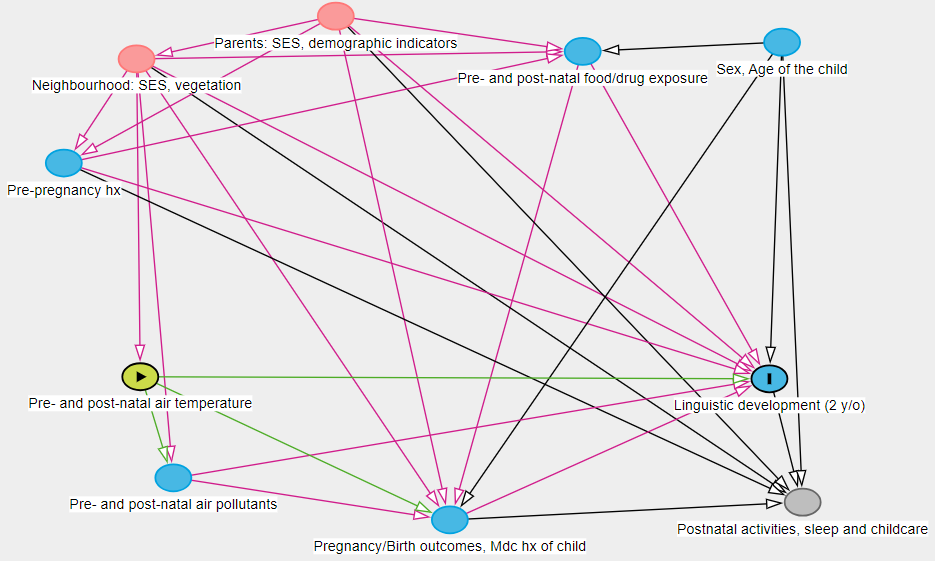


**B. Model considering "Pre- and post-natal food/drug exposure" as a mediating factor**

**
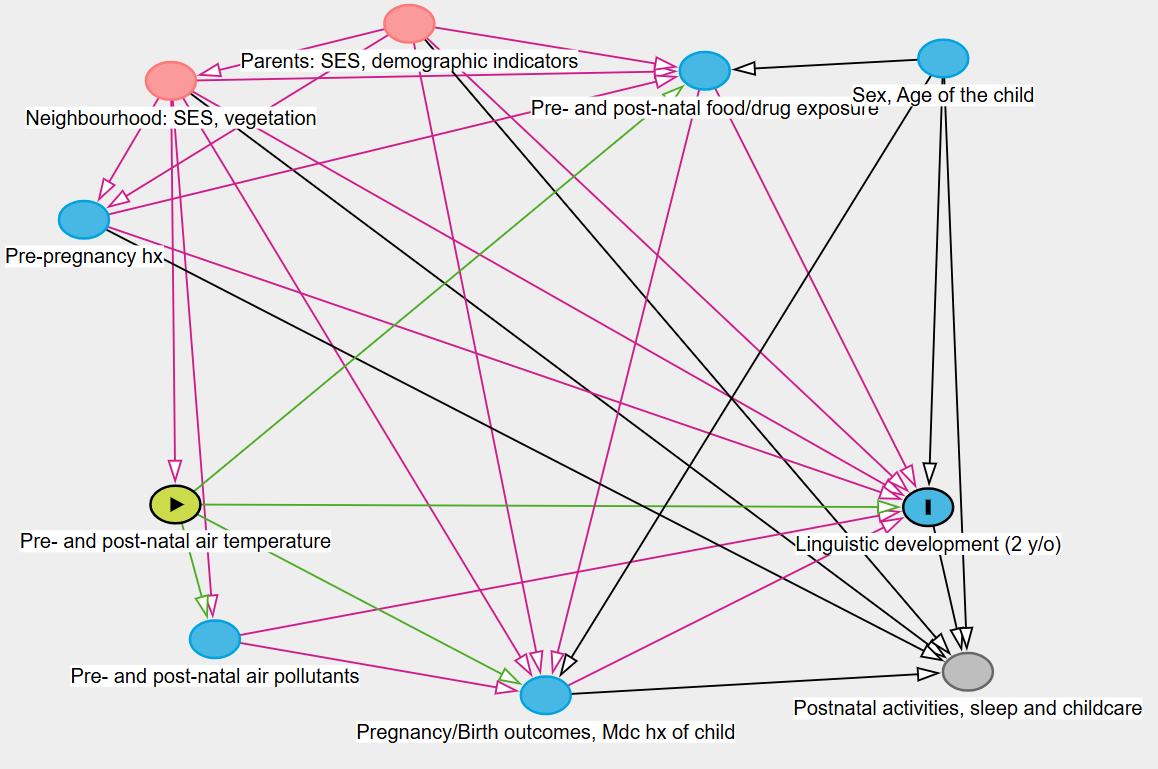
**

Description of the hypothetical relationship between exposure to air temperature and children’s linguistic development.

Legend. The green circle represents the exposure; the blue circle with a vertical bar represents the outcome; other blue circles represent ancestors of the outcome; pink circles represent ancestors of exposure and outcome.

Arrows represent relationships between variables; green arrows represent causal paths; pink arrows represent biasing paths.

SES, socio-economic status; Mdc, medical; hx, history; y/o, years old.

We did not adjust for factors that may be on the causal path between temperature and linguistic development. Nor did we adjust for factors that may be influenced by linguistic development in order to avoid a reverse causation bias. However, we did adjust for air pollution in secondary analyses in order to investigate the effects of ambient temperature above and beyond pollution.

This Directed Acyclic Graph (DAG) was created online (daggity.org).

# Supplementary Figure 2. Distribution of weekly overall temperatures during the study period.


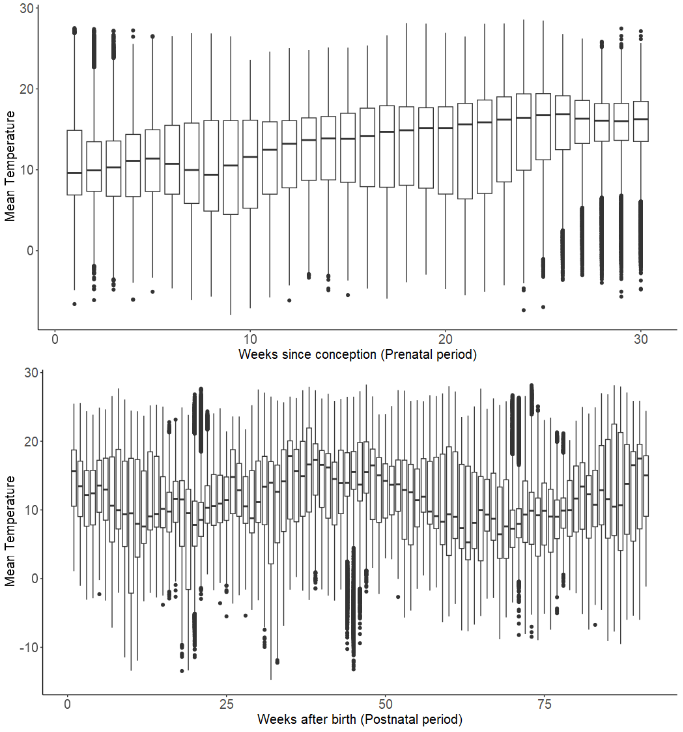


Upper panel: prenatal period; lower panel: postnatal period.

The top and the bottom of the boxes show the 25^th^ and the 75^th^ percentiles; the middle line inside the box indicates the median; the whiskers display the minimum and maximum values within 1.5 times the Inter-Quartile Range from the first and third quartiles; and the small circles represent the outliers.

# Supplementary Figure 3. Distribution of the MB-CDI score.


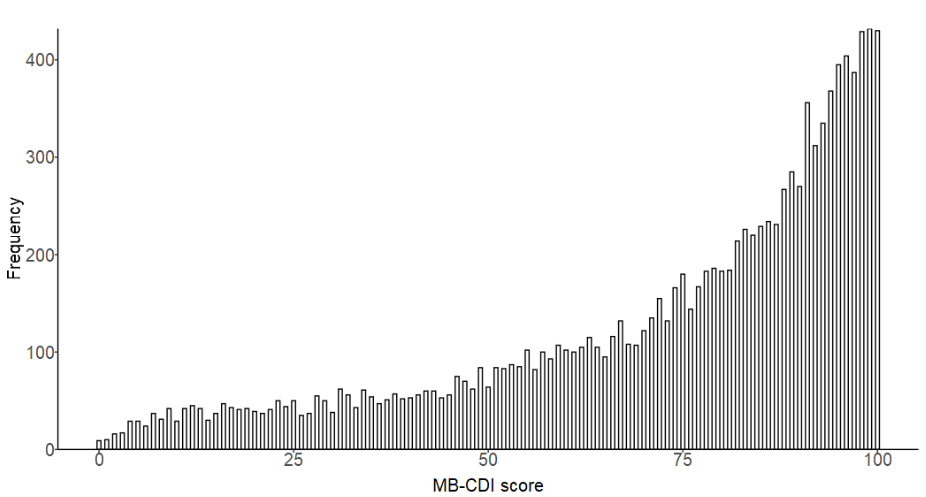


Legend. MB-CDI, MacArthur-Bates Communicative Development Inventories.

# **Supplementary Figure 4. Lag-specific effect of Heat on the MB-CDI score (from models adjusted for PM2.5).**


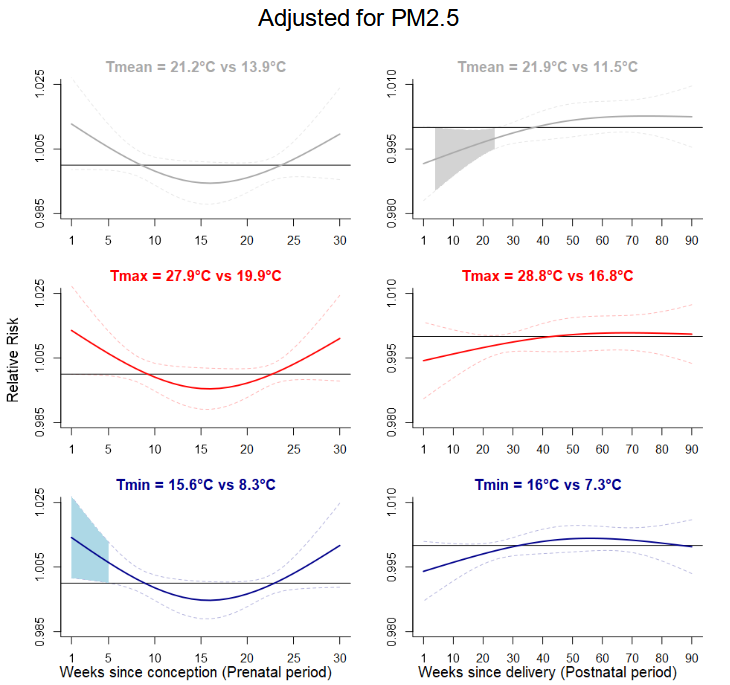


Adjusted relative risk (solid line) and 95% confidence interval (dashed lines) for the MB-CDI score associated with severe heat (95^th^ percentile vs. 50^th^ percentile) during the 30 weeks following conception (left) and the 91 first weeks of life (right). Risks lower than one indicate that higher temperatures, compared to the median temperature, are associated with a reduction in the MB-CDI score. Conversely, risks higher than one indicate that higher temperatures, compared to the median, are associated with an increase in the MB-CDI score.

Shaded areas indicate 95% confidence intervals that exclude one.

Upper panel: Overall temperature (Tmean); Middle panel: Daytime temperature (Tmax); Lower panel: Night-time temperature (Tmin).

Legend. MB-CDI, MacArthur-Bates Communicative Development Inventories; Wk, Week.

# **Supplementary Figure 5. Lag-specific effect of Heat on the MB-CDI score (from models adjusted for PM10).**


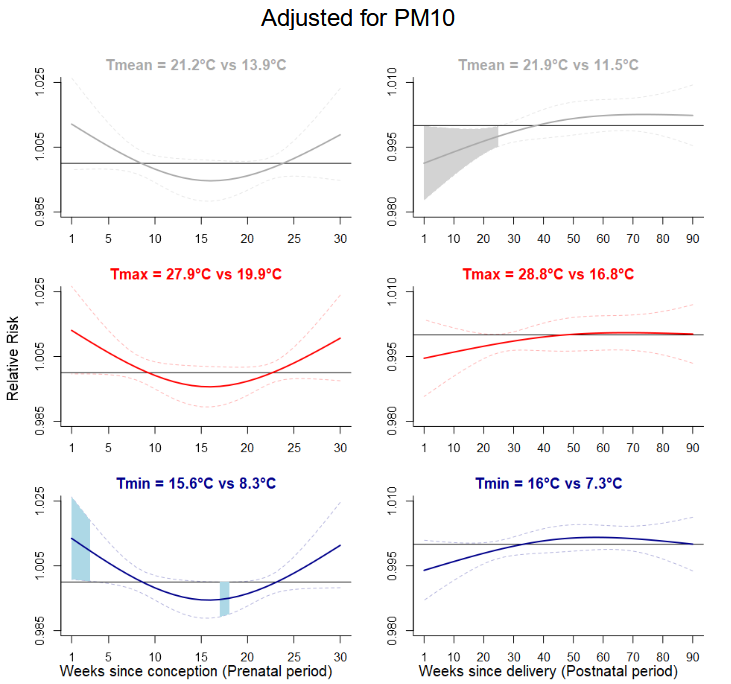


Adjusted relative risk (solid line) and 95% confidence interval (dashed lines) for the MB-CDI score associated with severe heat (95^th^ percentile vs. 50^th^ percentile) during the 30 weeks following conception (left) and the 91 first weeks of life (right). Risks lower than one indicate that higher temperatures, compared to the median temperature, are associated with a reduction in the MB-CDI score. Conversely, risks higher than one indicate that higher temperatures, compared to the median, are associated with an increase in the MB-CDI score.

Shaded areas indicate 95% confidence intervals that exclude one.

Upper panel: Overall temperature (Tmean); Middle panel: Daytime temperature (Tmax); Lower panel: Night-time temperature (Tmin).

Legend. MB-CDI, MacArthur-Bates Communicative Development Inventories; Wk, Week.

# **Supplementary Figure 6. Lag-specific effect of Heat on the MB-CDI score (from models adjusted for NO2).**


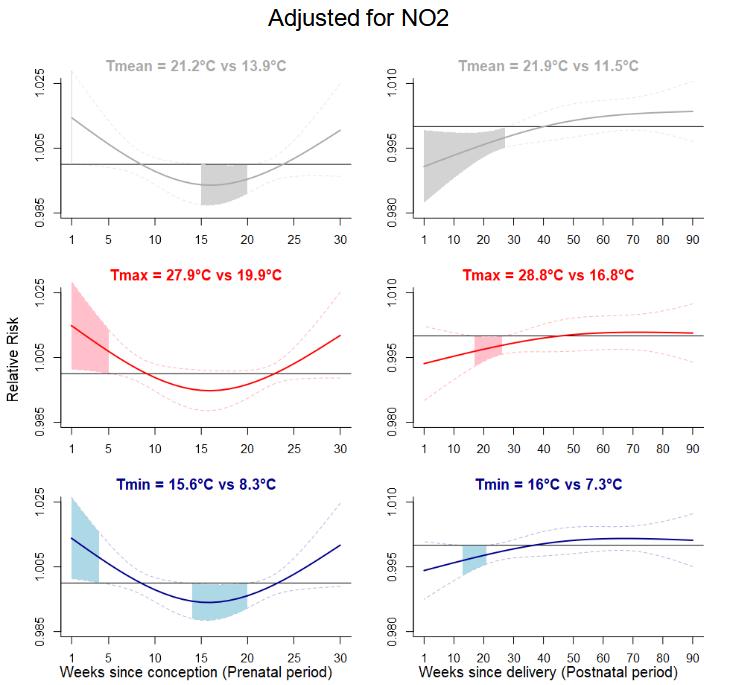


Adjusted relative risk (solid line) and 95% confidence interval (dashed lines) for the MB-CDI score associated with severe heat (95^th^ percentile vs. 50^th^ percentile) during the 30 weeks following conception (left) and the 91 first weeks of life (right). Risks lower than one indicate that higher temperatures, compared to the median temperature, are associated with a reduction in the MB-CDI score. Conversely, risks higher than one indicate that higher temperatures, compared to the median, are associated with an increase in the MB-CDI score.

Shaded areas indicate 95% confidence intervals that exclude one.

Upper panel: Overall temperature (Tmean); Middle panel: Daytime temperature (Tmax); Lower panel: Night-time temperature (Tmin).

Legend. MB-CDI, MacArthur-Bates Communicative Development Inventories; Wk, Week.

# **Supplementary Figure 7. Lag-specific effect of Cold on the MB-CDI score (from models adjusted for PM2.5).**


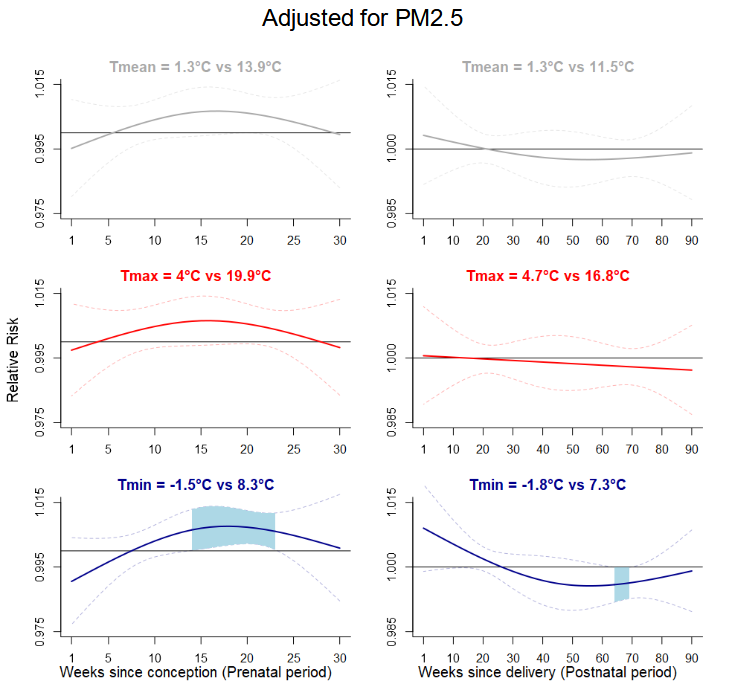


Adjusted relative risk (solid line) and 95% confidence interval (dashed lines) for the MB-CDI score associated with severe cold (5^th^ percentile vs. 50^th^ percentile) during the 30 weeks following conception (left) and the 91 first weeks of life (right). Risks lower than one indicate that colder temperatures, compared to the median temperature, are associated with a reduction in the MB-CDI score. Conversely, risks higher than one indicate that colder temperatures, compared to the median, are associated with an increase in the MB-CDI score.

Shaded areas indicate 95% confidence intervals that exclude one.

Upper panel: Overall temperature (Tmean); Middle panel: Daytime temperature (Tmax); Lower panel: Night-time temperature (Tmin).

Legend. MB-CDI, MacArthur-Bates Communicative Development Inventories; Wk, Week.

# **Supplementary Figure 8. Lag-specific effect of Cold on the MB-CDI score (from models adjusted for PM10).**


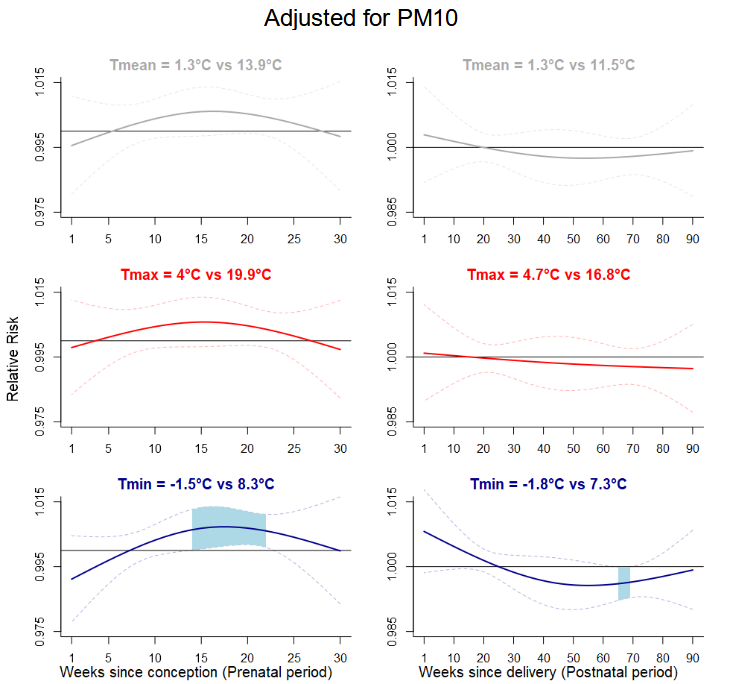


Adjusted relative risk (solid line) and 95% confidence interval (dashed lines) for the MB-CDI score associated with severe cold (5^th^ percentile vs. 50^th^ percentile) during the 30 weeks following conception (left) and the 91 first weeks of life (right). Risks lower than one indicate that colder temperatures, compared to the median temperature, are associated with a reduction in the MB-CDI score. Conversely, risks higher than one indicate that colder temperatures, compared to the median, are associated with an increase in the MB-CDI score.

Shaded areas indicate 95% confidence intervals that exclude one.

Upper panel: Overall temperature (Tmean); Middle panel: Daytime temperature (Tmax); Lower panel: Night-time temperature (Tmin).

Legend. MB-CDI, MacArthur-Bates Communicative Development Inventories; Wk, Week.

# **Supplementary Figure 9. Lag-specific effect of Cold on the MB-CDI score (from models adjusted for NO2).**


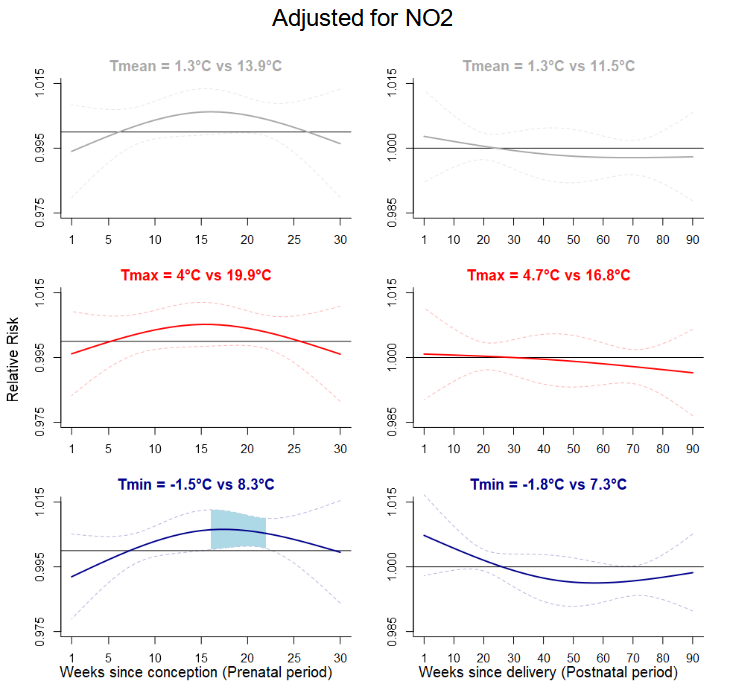


Adjusted relative risk (solid line) and 95% confidence interval (dashed lines) for the MB-CDI score associated with severe cold (5^th^ percentile vs. 50^th^ percentile) during the 30 weeks following conception (left) and the 91 first weeks of life (right). Risks lower than one indicate that colder temperatures, compared to the median temperature, are associated with a reduction in the MB-CDI score. Conversely, risks higher than one indicate that colder temperatures, compared to the median, are associated with an increase in the MB-CDI score.

Shaded areas indicate 95% confidence intervals that exclude one.

Upper panel: Overall temperature (Tmean); Middle panel: Daytime temperature (Tmax); Lower panel: Night-time temperature (Tmin).

Legend. MB-CDI, MacArthur-Bates Communicative Development Inventories; Wk, Week.

# **Supplementary Figure 10. Lag-specific effects of Heat on the MB-CDI score in infant boys and girls (from models unadjusted for pollution).**


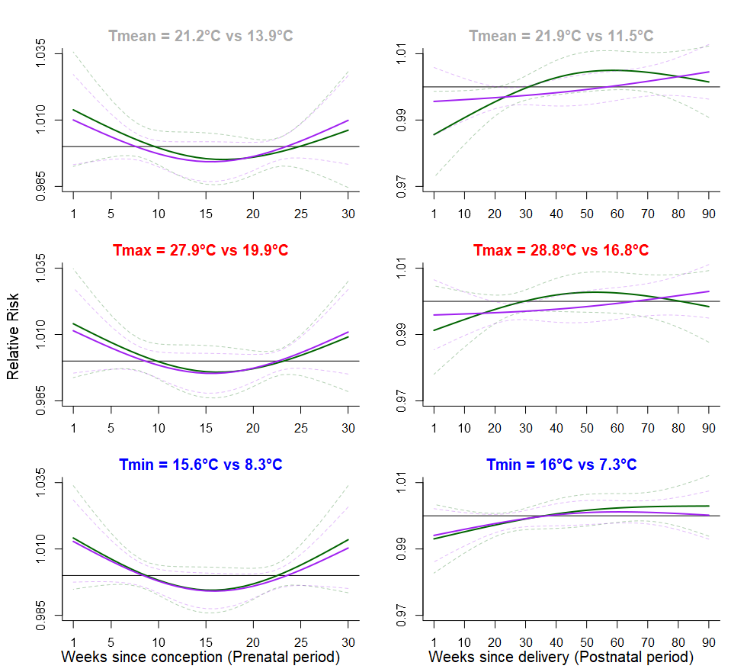


Adjusted relative risk (solid line) and 95% confidence interval (shaded area) for the MB-CDI score associated with severe heat (95^th^ percentile vs. 50^th^ percentile) during the 30 weeks following conception (left) and the 91 first weeks of life (right) in males (green) and females (purple). Risks lower than one indicate that higher temperatures, compared to the median temperature, are associated with a reduction in the MB-CDI score. Conversely, risks higher than one indicate that higher temperatures, compared to the median, are associated with an increase in the MB-CDI score.

There were no between-group differences.

Upper panel: Overall temperature (Tmean); Middle panel: Daytime temperature (Tmax); Lower panel: Night-time temperature (Tmin).

Legend. MB-CDI, MacArthur-Bates Communicative Development Inventories.

# **Supplementary Figure 11. Lag-specific effects of Cold on the MB-CDI score in infant boys and girls (from models unadjusted for pollution).**


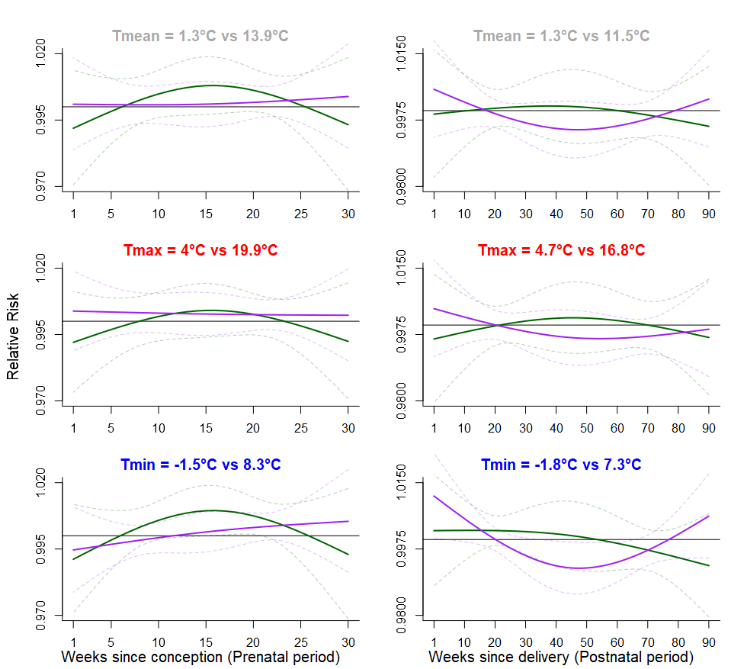


Adjusted relative risk (solid line) and 95% confidence interval (shaded area) for the MB-CDI score associated with severe cold (5^th^ percentile vs. 50^th^ percentile) during the 30 weeks following conception (left) and the 91 first weeks of life (right) in males (green) and females (purple). Risks lower than one indicate that lower temperatures, compared to the median temperature, are associated with a reduction in the MB-CDI score. Conversely, risks higher than one indicate that lower temperatures, compared to the median, are associated with an increase in the MB-CDI score.

There were no between-group differences.

Upper panel: Overall temperature (Tmean); Middle panel: Daytime temperature (Tmax); Lower panel: Night-time temperature (Tmin).

Legend. MB-CDI, MacArthur-Bates Communicative Development Inventories.

# References.

1. Attig M, Weinert S. What Impacts Early Language Skills? Effects of Social Disparities and Different Process Characteristics of the Home Learning Environment in the First 2 Years. Frontiers in Psychology [Internet]. 2020 [cited 2024 Jan 10];11. Available from: https://www.frontiersin.org/articles/10.3389/fpsyg.2020.557751

2. Carolan M, Frankowska D. Advanced maternal age and adverse perinatal outcome: A review of the evidence. Midwifery [Internet]. 2011 [cited 2024 Jan 10];27:793–801. Available from: https://www.sciencedirect.com/science/article/pii/S0266613810001208

3. Zweifel JE, Woodward JT. The risky business of advanced paternal age: neurodevelopmental and psychosocial implications for children of older fathers. Fertility and Sterility [Internet]. 2022 [cited 2024 Feb 6];118:1013–21. Available from: https://www.sciencedirect.com/science/article/pii/S0015028222019793

4. D’Onofrio BM, Rickert ME, Frans E, Kuja-Halkola R, Almqvist C, Sjölander A, et al. Paternal age at childbearing and offspring psychiatric and academic morbidity. JAMA Psychiatry. 2014;71:432–8.

5. Goisis A, Schneider DC, Myrskylä M. The reversing association between advanced maternal age and child cognitive ability: evidence from three UK birth cohorts. Int J Epidemiol [Internet]. 2017 [cited 2024 Jan 10];46:850–9. Available from: https://www.ncbi.nlm.nih.gov/pmc/articles/PMC5837600/

6. Kacenelenbogen N, Dramaix-Wilmet M, Schetgen M, Roland M, Godin I. Parental separation: a risk for the psychomotor development of children aged 28 to 32 months? A cross-sectional study. BMC Pediatr [Internet]. 2016 [cited 2024 Jan 10];16:89. Available from: https://doi.org/10.1186/s12887-016-0621-y

7. Clarke-Stewart KA, Vandell DL, McCartney K, Owen MT, Booth C. Effects of parental separation and divorce on very young children. Journal of Family Psychology. 2000;14:304–26.

8. Smith NR, Kelly YJ, Nazroo JY. Ethnic differences in cognitive development in the first 7 years: does maternal generational status matter? J Epidemiol Community Health. 2016;70:506–12.

9. Insee. Ethnic-based statistics [Internet]. 2016 [cited 2023 Nov 1]. Available from: https://www.insee.fr/en/information/2388586

10. Adélaïde L, Hough I, Seyve E, Kloog I, Fifre G, Launoy G, et al. Environmental and social inequities in continental France: an analysis of exposure to heat, air pollution, and lack of vegetation. J Expo Sci Environ Epidemiol. 2024;

11. Benz SA, Burney JA. Widespread Race and Class Disparities in Surface Urban Heat Extremes Across the United States. Earth’s Future [Internet]. 2021 [cited 2024 Jan 15];9:e2021EF002016. Available from: https://onlinelibrary.wiley.com/doi/abs/10.1029/2021EF002016

12. Klebanov PK, Brooks-Gunn J, Duncan GJ. Does Neighborhood and Family Poverty Affect Mothers’ Parenting, Mental Health, and Social Support? Journal of Marriage and Family [Internet]. 1994 [cited 2024 Jan 10];56:441–55. Available from: https://www.jstor.org/stable/353111

13. Pornet C, Delpierre C, Dejardin O, Grosclaude P, Launay L, Guittet L, et al. Construction of an adaptable European transnational ecological deprivation index: the French version. J Epidemiol Community Health. 2012;66:982–9.

14. Boukhabl M, Alkam D. Impact of Vegetation on Thermal Conditions Outside, Thermal Modeling of Urban Microclimate, Case Study: The Street of the Republic, Biskra. Energy Procedia [Internet]. 2012 [cited 2024 Jan 15];18:73–84. Available from: https://www.sciencedirect.com/science/article/pii/S1876610212007898

15. Dadvand P, Nieuwenhuijsen MJ, Esnaola M, Forns J, Basagaña X, Alvarez-Pedrerol M, et al. Green spaces and cognitive development in primary schoolchildren. Proc Natl Acad Sci U S A [Internet]. 2015 [cited 2024 Jan 15];112:7937–42. Available from: https://www.ncbi.nlm.nih.gov/pmc/articles/PMC4491800/

16. Dockx Y, Bijnens EM, Luyten L, Peusens M, Provost E, Rasking L, et al. Early life exposure to residential green space impacts cognitive functioning in children aged 4 to 6 years. Environ Int [Internet]. 2022 [cited 2024 Jan 15];161:107094. Available from: https://www.ncbi.nlm.nih.gov/pmc/articles/PMC8885429/

17. Grosjean F. Bilingual: Life and reality [Internet]. Harvard university press; 2010 [cited 2024 Aug 21]. Available from: https://www.degruyter.com/document/doi/10.4159/9780674056459-intro/html

18. Bjerkedal T, Kristensen P, Skjeret GA, Brevik JI. Intelligence test scores and birth order among young Norwegian men (conscripts) analyzed within and between families. Intelligence [Internet]. 2007 [cited 2024 Jan 10];35:503–14. Available from: https://www.sciencedirect.com/science/article/pii/S0160289607000062

19. Guo T, Wang Y, Zhang H, Zhang Y, Zhao J, Wang Y, et al. The association between ambient temperature and the risk of preterm birth in China. Sci Total Environ. 2018;613–614:439–46.

20. Shin EK, LeWinn K, Bush N, Tylavsky FA, Davis RL, Shaban-Nejad A. Association of Maternal Social Relationships With Cognitive Development in Early Childhood. JAMA Netw Open [Internet]. 2019 [cited 2024 Jan 10];2:e186963. Available from: https://www.ncbi.nlm.nih.gov/pmc/articles/PMC6484556/

21. Tong L, Kalish BT. The impact of maternal obesity on childhood neurodevelopment. J Perinatol. 2021;41:928–39.

22. Chen H, Qin L, Gao R, Jin X, Cheng K, Zhang S, et al. Neurodevelopmental effects of maternal folic acid supplementation: a systematic review and meta-analysis. Critical Reviews in Food Science and Nutrition [Internet]. 2023 [cited 2024 Jan 10];63:3771–87. Available from: https://doi.org/10.1080/10408398.2021.1993781

23. Key APF, Ferguson M, Molfese DL, Peach K, Lehman C, Molfese VJ. Smoking during Pregnancy Affects Speech-Processing Ability in Newborn Infants. Environmental Health Perspectives [Internet]. 2007 [cited 2024 Jan 10];115:623–9. Available from: https://ehp.niehs.nih.gov/doi/full/10.1289/ehp.9521

24. Gilman SE, Gardener H, Buka SL. Maternal Smoking during Pregnancy and Children’s Cognitive and Physical Development: A Causal Risk Factor? American Journal of Epidemiology [Internet]. 2008 [cited 2024 Jan 10];168:522–31. Available from: https://doi.org/10.1093/aje/kwn175

25. Lassen K, Oei TPS. Effects of maternal cigarette smoking during pregnancy on long-term physical and cognitive parameters of child development. Addictive Behaviors [Internet]. 1998 [cited 2024 Jan 10];23:635–53. Available from: https://www.sciencedirect.com/science/article/pii/S0306460398000227

26. Hendricks G, Malcolm-Smith S, Adnams C, Stein DJ, Donald KAM. Effects of prenatal alcohol exposure on language, speech and communication outcomes: a review longitudinal studies. Acta Neuropsychiatrica [Internet]. 2019 [cited 2024 Jan 10];31:74–83. Available from: https://www.cambridge.org/core/journals/acta-neuropsychiatrica/article/abs/effects-of-prenatal-alcohol-exposure-on-language-speech-and-communication-outcomes-a-review-longitudinal-studies/87157BFD79F1EB9E2B345BB1F36E1531

27. Galéra C, Bernard JY, van der Waerden J, Bouvard M-P, Lioret S, Forhan A, et al. Prenatal Caffeine Exposure and Child IQ at Age 5.5 Years: The EDEN Mother-Child Cohort. Biol Psychiatry. 2016;80:720–6.

28. Berglundh S, Vollrath M, Brantsæter AL, Brandlistuen R, Solé-Navais P, Jacobsson B, et al. Maternal caffeine intake during pregnancy and child neurodevelopment up to eight years of age—Results from the Norwegian Mother, Father and Child Cohort Study. Eur J Nutr [Internet]. 2021 [cited 2024 Feb 12];60:791–805. Available from: https://doi.org/10.1007/s00394-020-02280-7

29. Oken E, Radesky JS, Wright RO, Bellinger DC, Amarasiriwardena CJ, Kleinman KP, et al. Maternal Fish Intake during Pregnancy, Blood Mercury Levels, and Child Cognition at Age 3 Years in a US Cohort. American Journal of Epidemiology [Internet]. 2008 [cited 2024 Feb 12];167:1171–81. Available from: https://doi.org/10.1093/aje/kwn034

30. Daniels JL, Longnecker MP, Rowland AS, Golding J, Health TAST-U of BI of C. Fish Intake During Pregnancy and Early Cognitive Development of Offspring. Epidemiology [Internet]. 2004 [cited 2024 Feb 12];15:394. Available from: https://journals.lww.com/epidem/fulltext/2004/07000/fish_intake_during_pregnancy_and_early_cognitive.4.aspx

31. Huang Y, Iosif A-M, Hansen RL, Schmidt RJ. Maternal polyunsaturated fatty acids and risk for autism spectrum disorder in the MARBLES high-risk study. Autism [Internet]. 2020 [cited 2024 Feb 12];24:1191–200. Available from: https://doi.org/10.1177/1362361319877792

32. Horta BL, de Sousa BA, de Mola CL. Breastfeeding and neurodevelopmental outcomes. Current Opinion in Clinical Nutrition and Metabolic Care. 2018;21:174–8.

33. Part C, Filippi V, Cresswell JA, Ganaba R, Hajat S, Nakstad B, et al. How do high ambient temperatures affect infant feeding practices? A prospective cohort study of postpartum women in Bobo-Dioulasso, Burkina Faso. BMJ Open [Internet]. 2022 [cited 2025 Feb 21];12:e061297. Available from: https://www.ncbi.nlm.nih.gov/pmc/articles/PMC9535177/

34. Carnalla M, López-Olmedo N, Ramírez-Toscano Y, Cárdenas-Cárdenas LM, Canto-Osorio F, Rengifo-Reina H, et al. Binge drinking associated with mean temperature: a cross-sectional study among Mexican adults living in cities. Globalization and Health [Internet]. 2024 [cited 2025 Feb 21];20:29. Available from: https://doi.org/10.1186/s12992-024-01033-z

35. Momperousse D, Delnevo CD, Lewis MJ. Exploring the seasonality of cigarette‐smoking behaviour. Tob Control [Internet]. 2007 [cited 2025 Feb 21];16:69–70. Available from: https://www.ncbi.nlm.nih.gov/pmc/articles/PMC2598459/

36. Leaper C, Smith TE. A meta-analytic review of gender variations in children’s language use: talkativeness, affiliative speech, and assertive speech. Dev Psychol. 2004;40:993–1027.

37. Fiore AM, Naik V, Leibensperger EM. Air Quality and Climate Connections. Journal of the Air & Waste Management Association [Internet]. 2015 [cited 2024 Jan 15];65:645–85. Available from: https://doi.org/10.1080/10962247.2015.1040526

38. Buckley JP, Samet JM, Richardson DB. Commentary: Does Air Pollution Confound Studies of Temperature? Epidemiology [Internet]. 2014 [cited 2024 Jan 11];25:242. Available from: https://journals.lww.com/epidem/fulltext/2014/03000/Commentary___Does_Air_Pollution_Confound_Studies.14.aspx

39. Hough I, Sarafian R, Shtein A, Zhou B, Lepeule J, Kloog I. Gaussian Markov random fields improve ensemble predictions of daily 1 km PM2.5 and PM10 across France. Atmospheric Environment [Internet]. 2021 [cited 2024 Jan 3];264:118693. Available from: https://www.sciencedirect.com/science/article/pii/S135223102100515X

40. Barbalat G, Hough I, Dorman M, Lepeule J, Kloog I. A multi-resolution ensemble model of three decision-tree-based algorithms to predict daily NO2 concentration in France 2005–2022. Environmental Research [Internet]. 2024 [cited 2024 Aug 26];257:119241. Available from: https://www.sciencedirect.com/science/article/pii/S0013935124011460

41. Hoff-Ginsberg E, Shatz M. Linguistic input and the child’s acquisition of language. Psychological Bulletin. 1982;92:3–26.

42. Madigan S, McArthur BA, Anhorn C, Eirich R, Christakis DA. Associations Between Screen Use and Child Language Skills: A Systematic Review and Meta-analysis. JAMA Pediatrics [Internet]. 2020 [cited 2024 Jan 10];174:665–75. Available from: https://doi.org/10.1001/jamapediatrics.2020.0327

43. Dionne G, Touchette E, Forget-Dubois N, Petit D, Tremblay RE, Montplaisir JY, et al. Associations Between Sleep-Wake Consolidation and Language Development in Early Childhood: A Longitudinal Twin Study. Sleep [Internet]. 2011 [cited 2024 Jan 16];34:987–95. Available from: https://doi.org/10.5665/SLEEP.1148

44. Vissers C, Koolen S. Theory of Mind Deficits and Social Emotional Functioning in Preschoolers with Specific Language Impairment. Frontiers in Psychology [Internet]. 2016 [cited 2024 Jan 16];7. Available from: https://www.frontiersin.org/articles/10.3389/fpsyg.2016.01734

45. Bretherton L, Prior M, Bavin E, Cini E, Eadie P, Reilly S. Developing relationships between language and behaviour in preschool children from the Early Language in Victoria Study: implications for intervention. Emotional and Behavioural Difficulties [Internet]. 2014 [cited 2024 Jan 16];19:7–27. Available from: https://doi.org/10.1080/13632752.2013.854956

46. Longobardi E, Spataro P, Frigerio A, Rescorla L. Language and social competence in typically developing children and late talkers between 18 and 35 months of age. Early Child Development and Care [Internet]. 2016 [cited 2024 Jan 16];186:436–52. Available from: https://doi.org/10.1080/03004430.2015.1039529

47. McCabe PC. Social and behavioral correlates of preschoolers with specific language impairment. Psychology in the Schools [Internet]. 2005 [cited 2024 Jan 16];42:373–87. Available from: https://onlinelibrary.wiley.com/doi/abs/10.1002/pits.20064

48. Williams SM, Farmer VL, Taylor BJ, Taylor RW. Do More Active Children Sleep More? A Repeated Cross-Sectional Analysis Using Accelerometry. PLOS ONE [Internet]. 2014 [cited 2024 Jan 16];9:e93117. Available from: https://journals.plos.org/plosone/article?id=10.1371/journal.pone.0093117

49. Chang Z, Lei W. A Study on the Relationship Between Physical Activity, Sedentary Behavior, and Sleep Duration in Preschool Children. Frontiers in Public Health [Internet]. 2021 [cited 2024 Jan 16];9. Available from: https://www.frontiersin.org/articles/10.3389/fpubh.2021.618962

50. Barnett MA, Gustafsson H, Deng M, Mills-Koonce WR, Cox M. Bidirectional Associations Among Sensitive Parenting, Language Development, and Social Competence. Infant and child development [Internet]. 2012 [cited 2024 Aug 20];21:374. Available from: https://www.ncbi.nlm.nih.gov/pmc/articles/PMC4128493/

51. Dale PS, Tosto MG, Hayiou-Thomas ME, Plomin R. Why does parental language input style predict child language development? A twin study of gene–environment correlation. J Commun Disord [Internet]. 2015 [cited 2024 Aug 20];57:106–17. Available from: https://www.ncbi.nlm.nih.gov/pmc/articles/PMC4610950/

52. Schisterman EF, Cole SR, Platt RW. Overadjustment Bias and Unnecessary Adjustment in Epidemiologic Studies. Epidemiology. 2009;20:488–95.
